# Can human experts predict solubility better than computers?

**DOI:** 10.1186/s13321-017-0250-y

**Published:** 2017-12-13

**Authors:** Samuel Boobier, Anne Osbourn, John B. O. Mitchell

**Affiliations:** 1 Biomedical Sciences Research Complex and EaStCHEM School of Chemistry, University of St Andrews, St Andrews, KY16 9ST Scotland, UK; 2 Department of Metabolic Biology, John Innes Centre, Norwich Research Park, Norwich, NR4 7UH UK

## Abstract

In this study, we design and carry out a survey, asking human experts to predict the aqueous solubility of druglike organic compounds. We investigate whether these experts, drawn largely from the pharmaceutical industry and academia, can match or exceed the predictive power of algorithms. Alongside this, we implement 10 typical machine learning algorithms on the same dataset. The best algorithm, a variety of neural network known as a multi-layer perceptron, gave an RMSE of 0.985 log S units and an R^2^ of 0.706. We would not have predicted the relative success of this particular algorithm in advance. We found that the best individual human predictor generated an almost identical prediction quality with an RMSE of 0.942 log S units and an R^2^ of 0.723. The collection of algorithms contained a higher proportion of reasonably good predictors, nine out of ten compared with around half of the humans. We found that, for either humans or algorithms, combining individual predictions into a consensus predictor by taking their median generated excellent predictivity. While our consensus human predictor achieved very slightly better headline figures on various statistical measures, the difference between it and the consensus machine learning predictor was both small and statistically insignificant. We conclude that human experts can predict the aqueous solubility of druglike molecules essentially equally well as machine learning algorithms. We find that, for either humans or algorithms, combining individual predictions into a consensus predictor by taking their median is a powerful way of benefitting from the wisdom of crowds.

**Electronic supplementary material:**

The online version of this article (10.1186/s13321-017-0250-y) contains supplementary material, which is available to authorized users.

## Background

Solubility is the property of a chemical solute dissolving in a solvent to form a homogeneous system [1]. Solubility depends on the solvent used, as well as the pressure and temperature at which it was recorded. Water solubility is one of the key requirements of drugs, ensuring that they can be absorbed through the stomach lining and small intestine, eventually passing through the liver into the bloodstream. This means that low solubility is linked with poor bioavailability [2]. Another typical requirement of a drug is delivery in tablet form, again adequate solubility is needed. Tablets are strongly preferred to intravenous delivery of drugs, not least for patient compliance, ease of controlling the dose, and of self-administration. There are also toxicity problems associated with low solubility drugs, for example crystalluria caused by the drug forming a crystalline solid in the body [3]. Moreover, poor pharmacokinetics and toxicity are major causes of late stage failure in drug development. In fact 40% of drug failures stem from poor pharmacokinetics [4].

Prediction of key pharmaceutical properties has become increasingly important with the use of high throughput screening (HTS). As HTS has gained popularity, drug candidates have had increasingly higher molecular weight and lipophilicity, leading to lower solubility which is considered the predominant problem [5]. It is vital that solubility can be understood and predicted, in order to reduce the number of late stage failures due to poor bioavailability. Thus springs the need for ways to accurately predict both solubility and the essential properties, often referred to as ADMET (absorption, distribution, metabolism, elimination and toxicity), in which solubility is a key factor. As a way to increase the success of developing effective medicines, Lipinski’s popular “rule of five” was an empirical analysis of the attributes of successful drugs, giving guidelines on what makes a good pharmaceutical [2]. He found that effective drugs had molecular weight < 500, lipophilicity of log P < 5, and numbers of hydrogen bond donor and acceptor atoms that were < 5 and < 10 respectively. Increasingly, in silico approaches are being used to predict ADMET properties, in order to streamline the number of candidates coming through HTS.

Solubility itself is difficult to measure. Typically log S, the base 10 logarithm of the solubility as referred to units of mol/dm^3^, is reported. There are many different definitions of solubility and various experimental ways of measuring it, which can lead to poor reproducibility of solubility measurements. Thus with varied sources of data, especially when the exact details of the solubility methodology are not specified, assembling a high quality dataset for solubility prediction can be difficult. Thermodynamic solubility is the solubility measured under equilibrium conditions. It can be determined with a shake flask approach, or by using a method like CheqSol [6], where equilibration is speeded up by shuttling between super- and subsaturated solutions via additions of small titres of acid or alkali. The Solubility Challenge [7, 8] used its own bespoke dataset, measuring intrinsic aqueous thermodynamic solubility with the CheqSol method. Its authors reported high reproducibility and claimed random errors of only 0.05 log S units. Despite this, study of the literature suggests that overall errors in reported intrinsic solubilities of drug-like molecules are around 0.6–0.7 log S units, as discussed by Palmer & Mitchell and previously by Jorgensen & Duffy [9, 10].

This means that the best computational predictions possible would have root mean squared errors (RMSE) similar to the experimental error in reported solubilities. The feasible prediction accuracy will be dataset-dependent. Using various machine learning (ML) methods similar to those utilised herein, we obtained a best RMSE of 0.69 log S units for a test set of 330 druglike molecules, 0.90 for a different test set of 87 such compounds, 0.91 for the Solubility Challenge test set of 28 molecules, and in the same paper 1.11 log S units for a tenfold cross-validation of our DLS-100 set of 100 druglike compounds [11–15]. Further, we define a *useful* prediction as one with an RMSE smaller than the standard deviation of the experimental solubilities, to avoid being outperformed by the naïve assignment of the mean experimental solubility to all compounds [13].

The use of human participants is widespread in the social sciences, but remains a relatively unused tool in chemistry. This is largely due to the nature of the questions that chemists address. However, human experts are frequently used in surveys about the future of research areas in science, for example asking climate change experts to respond to a series of statements on the future of the field [16]. Similarly, the results of an expert survey on the future of artificial intelligence were published in 2016 [17].

The *wisdom of crowds* is the beneficial effect of recruiting many independent predictors to solve a problem [18, 19]. Over a 100 years ago, Galton described a competition at a country fair requiring participants to estimate the mass of a cow. Some guesses were large overestimates and others substantial underestimates. Nonetheless, the ensemble of estimates was able to make an accurate prediction, as reported in *Nature* [20]. If some predictors are likely to be very unreliable, then it is better to use the median estimate as the chosen prediction, avoiding the potentially excessive effect of a few ridiculous guesses on the mean [21]. In cheminformatics, the same kind of idea was exploited by Bhat et al. [22] to predict melting points, employing an ensemble of artificial neural networks rather than a crowd of humans. They reported a large improvement in accuracy, with the ensemble prediction being better than even the best performing single neural network. The use of multiple independent models is also fundamental to other ensemble predictors, such as Random Forest, and to consensus methods for rescoring docked protein–ligand complexes [23]. Utilising the wisdom of crowds requires an algorithm or experiment that can produce many independent predictors, though based on essentially the same pool of input data.

To our knowledge, there has only been one other recent study that has used human experts to solve chemical problems. Orphan drugs are potential pharmaceuticals that remain commercially undeveloped, often because they treat diseases too rare for commercially viable drug development by pharmaceutical companies in a competitive market. Regulators incentivise the development of these drugs by allowing market exclusivity; a new drug for these conditions is only approved if is judged to be sufficiently dissimilar to products already on the market. Judging whether or not two compounds are similar is time consuming for the team of experts. Thus, Franco et al. [24] asked whether computers could reproduce the rulings of experts. Human specialists were shown 100 pairs of molecules and asked to quantify their similarity. Their results were statistically compared to similarities computed with 2D fingerprint methods. The authors concluded that 2D fingerprint methods “can provide useful information” to regulatory authorities for judging molecular similarity.

Of considerable relevance to the current project is the Solubility Challenge [7, 8]. Recognising the difficulties in measuring solubility coupled with its vital importance to drug design, its authors reported the solubility of 100 drug-like molecules, with a high quality dataset and consistently using the CheqSol method [6]. The research community was then asked to submit predictions of the aqueous solubilities of 32 further compounds, which had been measured in-house, but were unreported. The subsequently reported results of the Challenge gave a measure of the state of the art in solubility prediction [8]. Nonetheless, the outcome of this blind challenge would have been more interesting and of greater utility had participants been asked to provide details of the computational methods, and of any experimental data beyond the training set, they used in their predictions.

Machine learning is a subset of Artificial Intelligence (AI), where models can change when exposed to new data [25]. In general, one can use the properties of a large training set to predict the properties of a typically smaller test set. Machine learning has found an array of applications from image recognition and intrusion detection to commercial use in data mining [26–28]. In chemistry, the application of machine learning is widespread in drug discovery and it can be used to detect toxicity, predict ADMET properties or derive Structure–Activity Relationships (SAR) [29–34]. Supervised machine learning problems can be divided into classification and regression. In classification models, the property to be predicted is a categorical variable and the prediction is which of these classes a new instance should be assigned to, such as *soluble* or *insoluble*. Regression problems deal with predicting continuous variables, for instance log S. In chemical applications of machine learning, the problem generally has two parts: firstly encoding molecular structure in the computer, and secondly finding an algorithmic or mathematical way of accurately mapping the encodings of structures to values or categories of the output property [21, 35]. The encoding typically consists of molecular descriptors, also known as features or attributes. There are thousands of descriptors in use, ranging from ones derived solely from the chemical structure, such as counts of atoms of each element in the molecule and topological and electronic indices, to experimentally derived quantities like log P or melting point [36].

## Methods

### Dataset and descriptors

Our dataset is the same set of 100 druglike molecules as used in our group’s previous work, which we call the DLS-100 set [13–15]. We chose this because it is a convenient set of high quality data for which we have a benchmark of the performance of other computational methods. Approximately two-fifths of the molecules had their solubilities measured with the CheqSol method [7, 8, 37, 38], and the remaining data were obtained from a small number of, so far as we can judge, generally reliable sources [39–43]. All our data are intrinsic aqueous solubilities, which correspond to the solubility of the neutral form only, in common with our previous studies of solubility [9, 11–13, 37, 44, 45]. For a few molecules, a somewhat arbitrary choice between slightly different quoted literature solubilities had to be made.

The molecules were split into two groups: 75 in the training set and 25 in the test set. The split was made with the following conditions: no molecule in the test set of the aforementioned Solubility Challenge would be in our test set, in case participants had been involved in the Solubility Challenge; the best known pharmacy drugs, like paracetamol, were placed in the training set; and the least soluble and most soluble single molecules were also placed in the training set to avoid any need for extrapolation. After these requirements had been satisfied, the rest of the split was chosen at random. Additional file 1 shows the names and structures of 75 compounds in the training set, their solubilities, and a literature source for the solubility; Additional file 2 does the same for the 25 test set molecules. Additional file 3 contains these data in electronic form (.xlsx), including SMILES.

We use SMILES (Simplified Molecular Input Line Entry System) to represent molecules for data input, with letters and numbers conveniently showing the connectivity of each atom, where the hydrogen atoms are not explicitly shown, for example Oc1ccccc1 is phenol [46, 47]. These SMILES strings are then used to compute the molecular descriptors which form the encoding of the molecular structure. For this study, we used exactly the same set of 123 Chemistry Development Kit (CDK) [48] descriptors as previously, re-using files originally obtained in 2012 rather than recalculating descriptors [13]. This ensures comparability with the prior work.

### Machine learning

Decision trees, named after the branching manner in which the algorithm is structured, make predictions based on a series of partitions of the data [49]. When a new instance is evaluated, it is directed along the branches of the tree according to its descriptor values and at each branch-point, called a node, takes one of the two possible routes, until it reaches a terminal leaf node. The decision tree’s prediction of the property value is then based on this partitioning of the data. If it is a category, such as *red* or *blue*, then a query instance will be assigned to a category according to the distribution of training instances at the relevant leaf. If the output property is a continuous variable, the regression model is based on the data at the leaf reached, and the tree is technically a regression tree. Splittings at the nodes are chosen to yield ever more homogeneous partitions and to minimise the entropy. This is implemented through the Gini impurity, a variant of the Gini index, which measures the entropy of the output properties of the instances [50]. Minimising this entropy favours trees which group together instances with similar property values at the same leaf node, as is highly desirable in making an accurate prediction.

The Random Forest (RF) machine learning method can be used either for classification or, as in the present work, for regression [51, 52]. RF leverages the wisdom of crowds by using a forest consisting of multiple stochastically different trees, each based on separately sampled datasets drawn from a common pool of data. Trees are grown based on recursive partitioning of training data consisting of multiple features for each object, the objects here being compounds. The trees are randomised firstly by being based on separate bootstrap samples of the data pool, samples of *N* out of *N* objects chosen with replacement. Secondly, trees are also randomised by being permitted to use only a stochastically chosen subset of the descriptors determined by a parameter known as *m*_*try*_, with a new subset of *m*_*try*_ descriptors being chosen at each node as the tree is built. For each node, a Gini-optimal [50] split is chosen, so that data are collected into increasingly homogeneous groups down the tree, and thus the set of molecules assigned to each terminal leaf node will share similar values of the property being predicted. The Random Forest thus has a number *ntree* of stochastically different trees, each derived from a fresh bootstrap sample of the training data. Such a Random Forest of regression trees can then be used to predict unseen numerical test data, with the predictions from the different trees being amalgamated by using their mean to generate the overall prediction of the forest.

Other tree-based ensemble predictors are also used in this work, and most of the above discussion applies equally to them. Bagging is another tree-based ensemble predictor [53]. As explained by Svetnick, Bagging is equivalent to RF with *m*_*try*_ equal to the total number of known descriptors, that is all descriptors are available for optimising the splitting at each node [52]. The Extra Trees (or Extremely randomised Trees) algorithm is also related to RF but a third level of randomisation is introduced in the form of the threshold for each decision being selected at random rather than optimised [54].

Ada Boost (Adaptive Boosting) [55] is another ensemble method also based, in our usage, on tree classifiers. The overall classifier is fitted to the dataset using a sequence of weak learners, which in this implementation are decision trees. The weights of each training set instance are equal to begin with, but with each cycle these weights are adjusted to optimise the classifier in the boosting process. Many such cycles are run and the model increasingly focuses on predicting the difficult cases; these potential outliers have greater influence here than in most other methods. The process by which the weights are optimised is a kind of linear regression, although the underlying weak learners here are not themselves linear.

Support Vector Machines (SVM) map data into high dimensional space. Kernel functions, typically non-linear, are used to map the data into a high dimensional feature space [56, 57]. An optimal hyperplane is constructed to separate instances of different classes, or in the current case of regression to play the role of a regression line. Put simply, the chosen hyperplane separates the instances such that the margins between the closest points on each side, called support vectors, are maximised. SVM is very effective for problems with many features to learn from, where the data have high dimensionality, and for sparse data [58].

K nearest neighbours (KNN) is a method for classification and regression, where the prediction is based on the property values of the closest training data instances [59]. For a test instance, the distance to each training instance in the descriptor space is calculated to identify its nearest neighbours; this is usually the Euclidean distance, though other metrics like the Manhattan distance are also usable. The values of different descriptors should be scaled if their ranges significantly differ. K refers to the number of neighbouring training instances to be considered in the prediction. In classification the prediction is the majority vote of K-nearest neighbours, whereas in regression the mean value of the target property amongst the K neighbours is taken.

Artificial Neural Networks (ANN) are inspired by the brain’s use of biological neurons, but are vastly less complex [60, 61]. A typical ANN will be simpler and smaller than the minimal 302 neuron brain of the nematode worm *C. elegans* [62]. The ANN architecture has neurons in both an input layer which receives the initial data and an output layer which relays the prediction of the target variable. Between the input and output layers lies a single hidden layer in the typical architecture, or alternatively multiple hidden layers in the case of Deep Learning. Each connection between neurons carries a weight, these being optimised during the training phase as the network learns how best to connect inputs and outputs. ANNs can suffer from overfitting and learning from noise, especially when the training set is small or varied [63]. The variant of ANN used in this study is a back-propagating Multi-Layer Perceptron (MLP) [64].

Projection to Latent Structures, or Partial Least Squares, (PLS) is a well-established method developed from multilinear regression [65]. PLS obtains a linear regression by projecting the input and output variables to a new space and addressing collinearity by reducing the number of variables, removing those which are least important for prediction. It is a simple method, but may be unsuitable for complex, and especially non-linear, problems.

Stochastic Gradient Descent (SGD) is another linear method, structuring the problem as a gradient-descent based minimisation of a loss function describing the prediction error. SGD optimises the hyperplane, a multidimensional analogue of a regression line, by minimising the loss function to convergence [66].

### Survey

The ethical approval from the School of Psychology and Neuroscience Ethics Committee, which acts on behalf of the University of St Andrews Teaching and Research Ethics Committee (UTREC), is more fully described under Declarations below. The ethics approval letter can be found in Additional file 4. The design of the survey was carefully planned in advance. The Qualtrics suite of software was used to create an online survey [67].

A human expert was defined as someone with chemistry expertise, working or studying in a university or industry. A total of 229 emailed invitations were sent out to identified experts. At the start of the survey, participants were asked their highest level of education and their current field of employment. A pop up link to a webpage with the training data was available on every screen: http://chemistry.st-andrews.ac.uk/staff/jbom/group/solubility/.

This is in essence an HTML version of Additional file 1. The training data were displayed in a random order, with their respective log S values. Participants were then shown each molecule in the test set, in a random order. They were then asked to predict the aqueous solubility based on the training data. The molecules were displayed as skeletal formulae, drawn with the program ChemDoodle [68]. All molecules were shown at the same resolution. A full copy of the survey can be found in Additional file 5.

## Results

### Choice of median-based consensus predictors

Our simple experimental design provides no basis to pick either a best machine learning method or best human predictor before analysing the test set results. Hence, we decided in advance that our consensus machine learning predictor would be based on the median solubility predicted for each molecule amongst the ten algorithms. Similarly, our best human predictor would be based on the median solubility predicted for each molecule amongst the human participants. This selection of ensemble models means that we expect both our chosen consensus predictors to benefit from the wisdom of crowds [18–20]. We also examine the post hoc best individual machine learning method and best human predictor.

### Machine learning algorithms

The ten machine learning algorithms were trained on the training set and run on each of the 25 molecules of the test set to generate the predicted solubility. These computed solubilities are shown in Additional file 6; their standard deviation was 1.807 log S units. We assessed each machine learning method in terms of the root mean squared error (RMSE), average absolute error (AAE), coefficient of determination which is the square of the Pearson correlation coefficient (R^2^), Spearman rank correlation coefficient (ρ), and number of correct predictions within a margin of one log S unit (NC). These results are shown in Table 1.

**Table 1:** Statistical measures of the performance of the 10 machine learning algorithms and the median-based machine learning consensus predictor

	RMSE	R^2^	ρ	NC	AAE
MLP	0.985	0.706	0.837	19	0.728
RF	1.165	0.583	0.736	20	0.802
Bagging	1.165	0.583	0.726	20	0.803
KNN	1.204	0.540	0.704	15	0.917
ExtraTrees	1.227	0.542	0.728	18	0.837
AdaBoost	1.235	0.545	0.708	19	0.851
PLS	1.265	0.507	0.670	15	0.980
SVM	1.280	0.520	0.694	16	0.925
SGD	1.429	0.577	0.752	11	1.185
Decision tree	1.813	0.260	0.530	17	1.198
ML median	1.140	0.601	0.762	18	0.778

We assessed each machine learning method in terms of the root mean squared error (RMSE), coefficient of determination—which is the square of the Pearson correlation coefficient (R^2^), Spearman rank correlation coefficient (ρ), number of correct predictions within a margin of one log S unit (NC), and average absolute error (AAE)

The MLP performed best of the machine learning algorithms on RMSE, AAE, R^2^, and ρ. Its RMSE of 0.985 log S units is encouraging and its R^2^ of 0.706 is a decent result for this dataset, although the different validation methods mean that comparisons with McDonagh et al. [13] can be no more than semi-quantitative. RF also produces good results, with an RMSE of 1.165, and ranks in the top three individual machine learning predictors on all criteria. Alongside the closely related Bagging method, RF is one of two algorithms to obtain the highest number of correct predictions, with 20. The ten machine learning predictors spanned a range of RMSE from 0.985 to 1.813 log S units. The worst RMSE came from a single decision tree and was essentially identical to the standard deviation (SD) of the test set solubilities; the remaining nine methods gave prediction RMSE well below the sample SD, and thus fulfilled the usefulness criterion.

Our consensus ensemble median machine learning predictor beat nine of the ten individual algorithms on each of RMSE, AAE, R^2^ and ρ. However, MLP in fact outperformed it on each of these measures and is post hoc clearly the best ML algorithm. Paired difference t-tests on the prediction errors, described in detail below, show few statistically significant differences in the performance of the ML predictors. The only such instances of significance are that the consensus predictor is significantly different from PLS and SGD at the 5% level, and MLP is also significantly different from SGD. Machine learning scripts used are given in Additional file 7.

### Human predictors

A total of 22 answer sets were received from human participants, a response rate of 9.6%. Of the participants, four were professional PhD holding scientists working in industry, one PhD engaged in scientific communications, eight PhD holders working as University academics between postdoctoral and professorial level, four current postgraduate students, and five current undergraduate students. Amongst the submissions was a set of predictions by a self-identified software developer, each solubility being quoted to five decimal places. We considered that the circumstantial evidence of computer use was sufficiently strong to exclude these predictions from the human predictors. Interestingly, these predictions performed almost identically to those from our post hoc best ML method, which was MLP. The software developer’s results achieved a statistically significant performance difference compared with the ML method SGD in the paired difference t-tests. By design, they did not contribute to the ML consensus predictor. The exclusion of the software developer’s predictions from the human expert section of the study left a total of 17 participants who made a prediction for each of the 25 molecules, and four who predicted a subset. These estimated solubilities are shown in Additional file 8.

The two best sets of human responses were from anonymous respondents identified as participants 11 and 7, both recorded as being as PhD holders working in academic research. Participant 11 generated an RMSE of 0.942 log S units and an R^2^ of 0.723, ranking first by RMSE, R^2^, NC and AAE amongst the individual participants. This performance included 18 correct predictions, more than any other individual human entrant. Participant 7 achieved an RMSE of 1.187 log S units, an R^2^ of 0.637, and 17 correct predictions, while also ranking the compounds best with a ρ value of 0.867. The 17 full responses achieved RMSE values spanning a large range from 0.942 to 3.020 log S units. Of these, eight were considered clearly useful with RMSE values well below the sample SD; four were close to the SD, within a range of ± 0.15 units of it; five were beyond this range and considered not to qualify as useful predictors on the usual criterion. Nonetheless, all the complete sets of predictions were correct to within one log unit for at least nine molecules.

Our consensus ensemble median-based human predictor was constructed by taking the median of all human predictions received for each compound, including those from partial entries. This predictor performed very well, scoring an RMSE of 1.087 and an R^2^ of 0.632, respectively beaten by only one and two individual humans. The median human predictor made 21 correct predictions and achieved an AAE of 0.732 log S units, both better than any individual. Using a paired difference *t* test methodology on the absolute errors of each method, described in detail below, we find that the differences between this consensus predictor and 13 of the 17 human experts are statistically significant at the 5% level. Among the human predictions, 24 of the 136 pairwise comparisons show statistically significant differences at the 5% level. Thus there is substantially more variation in the quality of human predictions than of ML predictions.

### Comparison of consensus median-based machine learning and human predictors

An overall comparison of the median-based consensus ML and human descriptors is given in Table 2. While the consensus human classifier performs slightly better on each of the five measures, we need to establish whether the difference between the two is statistically significant. To do this, we carry out a paired difference test. For each compound, we consider the absolute error made by the consensus predictors, regardless of whether the predictions were underestimates or overestimates of the true solubility. These are shown in Table 3, with the difference indicated as positive if the human classifier performs better, and negative if the machine learning one is more accurate for that molecule. The paired difference test seeks to establish whether there is a significant difference in the performance of the two classifiers over the test set of 25 compounds. Thus, we estimate the p value, the probability that so great a difference could arise by chance under the null hypothesis that the two classifiers are of equal quality. Using the data in Table 3, we have carried out both a paired difference *t* test and also Menke and Martinez’s permutation test [69]. These tests produced p values of 0.576 for the Student’s *t* test and 0.575 for the permutation test, which show clearly that there is no statistically significant difference between the power of the two classifiers. The relative smallness of a test set containing 25 molecules somewhat limits the statistical power of such a comparison. However, during testing of the survey we found that predicting solubilities for larger sets of compounds became a long and onerous task, likely to prove beyond the patience of participants. The test set size and methodology is also sufficient to identify significant differences between individual human predictors; out of the 153 pairwise *t* tests amongst these 18 predictors, including the consensus one, 37 are significant at the 5% level.

**Table 2:** Comparison of statistical measures of the performance of the median-based machine learning consensus predictor and the median-based human consensus predictor in terms of the root mean squared error (RMSE), coefficient of determination—which is the square of the Pearson correlation coefficient (R^2^), Spearman rank correlation coefficient (ρ), number of correct predictions within a margin of one log S unit (NC), and average absolute error (AAE)

	Median-based ML	Median-based human
RMSE	1.140	1.087
R^2^	0.601	0.632
ρ	0.762	0.817
NC	18	21
AAE	0.778	0.732

**Table 3:** Performance of median-based consensus classifiers, errors are absolute (unsigned) and are measured in log S units

Compound	ML error	Human error	Difference
4-Aminobenzoic acid	0.07	0.13	− 0.06
4-Aminosalicylic acid	0.23	0.76	− 0.53
Antipyrine	3.73	2.98	0.75
Chloramphenicol	0.35	0.39	− 0.04
Corticosterone	0.11	0.06	0.05
Dapsone	0.54	0.29	0.25
Primidone	0.06	0.14	− 0.08
Estrone	0.87	0.82	0.05
Alclofenac	0.30	0.12	0.18
5-Fluorouracil	0.46	0.62	− 0.16
Griseofulvin	0.44	0.25	0.19
Fluometuron	0.53	0.04	0.49
Fluconazole	1.09	0.70	0.39
Khellin	0.17	0.98	− 0.81
Clozapine	1.37	0.71	0.66
Norethisterone	0.63	0.63	0.00
Nicotinic acid	0.58	0.35	0.23
Perphenazine	0.16	0.16	0.00
Pteridine	2.22	3.02	− 0.80
Salicylamide	0.23	0.49	− 0.26
Sulfanilamide	0.54	0.14	0.40
Gliclazide	1.03	0.80	0.23
Trihexyphenidyl	1.98	1.45	0.53
Triphenylene	0.15	0.27	− 0.12
Mifepristone	1.57	2.00	− 0.43
Average	0.778	0.732	0.046

The difference is meaningfully signed, with a positive value where the human median-based classifier performed better on that compound and a negative value where the machine learning median-based classifier performed better

### Comparison of best machine learning and human predictors

While the identities of the best individual machine learning and human predictors were only known after the fact, it is nonetheless of interest to identify and compare them. Given the nature of the statistical measure we are using, for this comparison we select the individual classifiers with the lowest AAE over the 25 molecules. In each case, these are the same classifiers that have the lowest RMSE and the highest R^2^, the multi-layer perceptron (MLP) and human participant 11. Their overall performance data are shown in Table 4, with the per-compound comparison in Table 5. The differences are small, though one might observe that the human performs better on three criteria and the perceptron on two.

**Table 4:** Comparison of statistical measures of the performance of the best single machine learning predictor and the best individual human predictor

	Multi-layer perceptron	Human participant 11
RMSE	0.985	0.942
R^2^	0.706	0.723
Spearman ρ	0.837	0.853
Number correct	19	18
AAE	0.728	0.734

**Table 5:** Performance of best individual classifiers, errors are absolute (unsigned) and are measured in log S units

Compound	MLP error	Human 11 error	Difference
4-Aminobenzoic acid	0.42	0.63	− 0.21
4-Aminosalicylic acid	0.39	0.04	0.35
Antipyrine	1.90	1.48	0.42
Chloramphenicol	0.78	0.89	− 0.11
Corticosterone	0.00	0.76	− 0.76
Dapsone	0.41	0.09	0.32
Primidone	1.45	0.36	1.09
Estrone	0.78	1.32	− 0.54
Alclofenac	0.02	1.13	− 1.11
5-Fluorouracil	0.07	0.97	− 0.90
Griseofulvin	0.90	1.25	− 0.35
Fluometuron	0.33	0.46	− 0.13
Fluconazole	0.22	0.20	0.02
Khellin	0.13	0.02	0.11
Clozapine	0.33	0.76	− 0.43
Norethisterone	1.53	0.37	1.16
Nicotinic acid	0.59	0.15	0.44
Perphenazine	0.53	0.84	− 0.31
Pteridine	1.00	0.02	0.98
Salicylamide	0.23	1.34	− 1.11
Sulfanilamide	0.71	0.14	0.57
Gliclazide	1.30	0.29	1.01
Trihexyphenidyl	2.93	2.20	0.73
Triphenylene	0.38	0.73	− 0.35
Mifepristone	0.86	1.90	− 1.04
Average	0.728	0.734	− 0.005

The difference is meaningfully signed, with a positive value where the best human classifier performed better on that compound and a negative value where the best machine learning classifier performed better

The statistical significance tests led to p-values of 0.970 for the t-test and 0.969 for the permutation test, indicating that there is no statistically significant difference between the power of the two classifiers.

### Human predictors: data issues

#### Possible data input ambiguity

Given that 74 out of 75 compounds in the training set and 23 out of 25 in the test set have negative log S values, we expected that the overwhelming majority of predictions would be of negative log S values. Making a prediction of a negative value requires the participant to type a minus sign as part of their input. In fact, a modest number of unexpected individual positive predictions were made. Some of these appear to be clear mistakes; there are three predictions of log S between 4.1 and 6.0 for molecules where the median predictions were between − 3.25 and − 4.5. One human participant subsequently contacted us to report having made at least one sign error. Five other positive valued predictions of log S between 0.2 and 3.2 may or may not be intentional.

While the results analysed above are for the data in their original unedited state, we have also considered the effect of ‘correcting’ for likely data errors. In that analysis alone, we have swapped the signs of any predictions of positive log S values where this would reduce the error; this means that all such predictions for compounds with negative experimental log S values had their signs provisionally changed.

The principal effect of this change would be to improve some of the weaker human predictors. In this adjusted set of results, the 17 full responses achieved RMSE values spanning a smaller range from 0.942 to 2.313 log S units. Of these, nine were considered clearly useful with RMSE values well below the sample SD; five were within a range of ± 0.15 units of the SD; three were beyond this range and considered not to be useful predictors on the usual criterion. All the complete sets of human predictions were correct for at least ten molecules. Although the range of prediction quality is reduced, we still note significant differences in predictive power between classifiers even when suspect human predictions are sign-reversed; out of the 153 pairwise t-tests amongst these 18 predictors, including the consensus one, 35 are significant at the 5% level.

The median-based approach to constructing our consensus classifiers is deliberately designed to be robust to the presence of outlying individual predictions. Although five of the median predictions change slightly upon adjustment of suspect signs, the overall statistics are barely affected with a new RMSE of 1.083, R^2^ of 0.639, ρ of 0.809, 21 correct predictions, and an AAE of 0.735. The comparison with the consensus machine learning classifier is hardly altered, with a p value from the paired difference *t* test of 0.600. Since the identity and statistics of the best human classifier are unaffected, the comparison of the best individual classifiers remains the same under the sign swaps. Thus we note that swapping signs of putative accidental positive log S predictions would have no effect on the main results of this paper, but would improve some of the weaker-performing human classifiers. We do not consider it further.

#### Data issues in survey training set

At a late stage, it was unfortunately discovered that the data used in the survey training set corresponded to an earlier draft, not the final version, of the solubilities used by McDonagh et al. [13]. There were non-trivial (> 0.25 log S units) differences between the solubility used in the survey training set and the published DLS-100 solubility for six compounds. For sulindac, the DLS-100 solubility is − 4.50 taken from Llinas et al. [7], but the survey training value was − 5.00 from Rytting et al. [39], for L-DOPA, the DLS-100 solubility value is − 1.82 taken from Rytting et al. [39], while the provisional value used in survey training was − 1.12; for sulfadiazine the DLS-100 solubility value is − 3.53 originally taken from Rytting et al. [39], while the value used in survey training was − 2.73; for guanine the DLS-100 solubility is − 4.43 taken from Llinas et al. [7], but the survey training value was the − 3.58 from Rytting et al. [39]; for cimetidine the correct DLS-100 solubility is − 1.69 taken from Llinas et al. [7], but an erroneous survey training value of − 3.60 was used. For the remaining 70 training set molecules, the DLS-100 and survey training set solubilities are either identical or within 0.25 log S units.

While it was not feasible to repeat the survey, it is possible to train the machine learning algorithms on both sets of training data. The main machine learning results reported herein are based on the correct DLS-100 solubilities, but we have also explored the effect of using the imprecise provisional data from the survey training. The effects of the machine learning results are small, though strangely the imprecise training data led to very slightly better median machine learning classifier results (RMSE = 1.095, R^2^ = 0.632). It appears that the data differences between the survey training set and the DLS-100 set had very little effect on the quality of the machine learning predictions and therefore are unlikely to have had a substantial effect on the human predictions.

### Predictions for different compounds

**Fig. 1:**
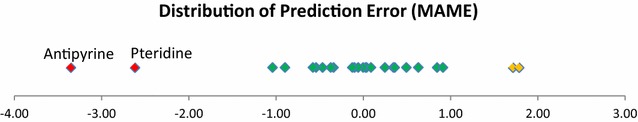
Per-compound distribution of the average of the absolute prediction errors of the two consensus predictors (Mean Absolute Median Error, MAME) for the test set. Compounds with errors more than one standard deviation above or below the mean signed error are in orange, those more than two standard deviations away are in red

**Fig. 2:**
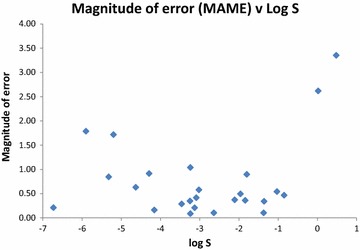
Magnitude of the average of the absolute prediction errors of the two consensus predictors (Mean Absolute Median Error, MAME) plotted against experimental log S for the 25 compounds in the test set

**Fig. 3:**
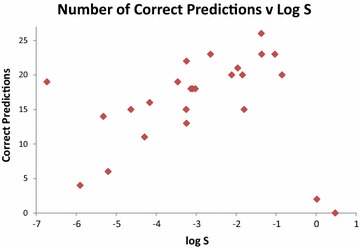
Number of correct predictions within ± 1 log S unit recorded for each molecule from 27 predictors (10 machine learning, 17 human), plotted against experimental log S

There is a substantial variation in prediction accuracy between the different molecules in our dataset. In Table 6 below, we rank the 25 compounds by the sizes of the errors from the two consensus predictors. For each compound, we define the average of these two unsigned consensus errors as the Mean Absolute Median Error (MAME), which we display in Fig. 1, and also alongside the number of correct predictions in Table 6. Figure 2 shows the MAME as a function of log S, and Fig. 3 illustrates how the number of correct predictions varies with solubility. As a general trend, compounds with solubilities around the middle of the range are well-predicted. The two most soluble molecules, pteridine and antipyrine, are the two worst predicted according to both measures. For the least soluble compounds, the picture is mixed. The second and fourth most insoluble compounds, mifepristone and trihexyphenidyl, are poorly handled, being the third and fourth worst predicted on either measure. However, the most insoluble compound, triphenylene, is well predicted and the third most insoluble, estrone, moderately well predicted with 14 correct predictions.

**Table 6:** The 25 test set compounds ranked by the average of the absolute prediction errors of the two consensus predictors (mean absolute median error, MAME)

Compound	Log S	ML median	Error	Human median	Error	MAME	NC
Corticosterone	− 3.24	− 3.13	0.11	− 3.30	− 0.06	0.09	22
4-Aminobenzoic acid	− 1.37	− 1.44	− 0.07	− 1.50	− 0.13	0.10	26
Primidone	− 2.64	− 2.70	− 0.06	− 2.50	0.14	0.10	23
Perphenazine	− 4.16	− 4.32	− 0.16	− 4.00	0.16	0.16	16
Alclofenac	− 3.13	− 2.83	0.30	− 3.25	− 0.12	0.21	18
Triphenylene	− 6.73	− 6.58	0.15	− 7.00	− 0.27	0.21	19
Fluometuron	− 3.46	− 2.93	0.53	− 3.50	− 0.04	0.29	19
Sulfanilamide	− 1.36	− 1.90	− 0.54	− 1.50	− 0.14	0.34	23
Griseofulvin	− 3.25	− 2.81	0.44	− 3.00	0.25	0.35	15
Salicylamide	− 1.84	− 1.61	0.23	− 1.35	0.49	0.36	20
Chloramphenicol	− 2.11	− 2.46	− 0.35	− 2.50	− 0.39	0.37	20
Dapsone	− 3.09	− 3.63	− 0.54	− 2.80	0.29	0.42	18
Nicotinic acid	− 0.85	− 1.43	− 0.58	− 1.20	− 0.35	0.47	20
4-Aminosalicylic acid	− 1.96	− 1.73	0.23	− 1.20	0.76	0.49	21
5-Fluorouracil	− 1.03	− 1.49	− 0.46	− 1.65	− 0.62	0.54	23
Khellin	− 3.02	− 3.19	− 0.17	− 4.00	− 0.98	0.58	18
Norethisterone	− 4.63	− 4.00	0.63	− 4.00	0.63	0.63	15
Estrone	− 5.32	− 4.45	0.87	− 4.50	0.82	0.85	14
Fluconazole	− 1.80	− 2.89	− 1.09	− 2.50	− 0.70	0.90	15
Gliclazide	− 4.29	− 3.26	1.03	− 3.49	0.80	0.91	11
Clozapine	− 3.24	− 4.61	− 1.37	− 3.95	− 0.71	1.04	13
Trihexyphenidyl	− 5.20	− 3.22	1.98	− 3.75	1.45	1.72	6
Mifepristone	− 5.90	− 4.33	1.57	− 3.90	2.00	1.79	4
Pteridine	0.02	− 2.20	− 2.22	− 3.00	− 3.02	2.62	2
Antipyrine	0.48	− 3.25	− 3.73	− 2.50	− 2.98	3.35	0

As noted before, many compounds have multiple and sometimes significantly different solubility values reported in the literature [9]. There can be confusion between different definitions of solubility, inclusion or exclusion of ionised forms, ambiguity between polymorphs, systematic differences between experimental methods, kinetic solubility may be wrongly identified as thermodynamic, or the solubility of the wrong compound may be measured due to unanticipated chemical reactions occurring in the experimental set-up. While we do not claim that poor predictability always implies likely error in the experimental value, this is not unknown. In the Solubility Challenge, indomethacin was very poorly predicted by computational methods compared to other compounds of similar reported solubility [8]. Subsequent investigation by Comer et al. demonstrated that indomethacin had undergone hydrolysis during the original CheqSol experiment, and gave a corrected value for its intrinsic solubility [70].

In the distribution of signed errors in the present dataset, only two compounds produce errors (MAME) which lie more than two standard deviations away from the mean; these are antipyrine and pteridine (Fig. 1). For antipyrine, the worst predicted molecule in our dataset, the intrinsic aqueous solubility we used is log S = 0.48, as listed in the dataset curated by Rytting et al. [39] on the basis of earlier work by Herman and Veng-Pedersen [71]. An alternative value of log S = − 0.56 originates from the Aquasol database [72]. Even using this alternative value, antipyrine would still be among the two or three worst predicted compounds in our study. Yalkowsky et al. [73] list seven different room temperature log S values for antipyrine, ranging from − 0.66 to 0.55, their annotations suggesting that they believe the higher solubilities to be more accurate. For pteridine, the solubility of log S = 0.02 was measured by CheqSol and reported in Palmer et al. [37] Two reports from Albert et al. in the 1950’s of solubility of “one part pteridine with 7.2 or 7 parts water” have been translated by Yalkowsky et al. into molar units to give log S = − 0.02 and − 0.03, respectively, and hence barely differ from the CheqSol result [73–75].

## Discussion

We have carried out a comparison of consensus predictors from machine learning algorithms and from human experts, the predictors being constructed so as not to require prior selection of the algorithm or human expected to obtain the best results. Comparing over a number of statistical measures of accuracy, we find that there is very little difference in prediction quality between the machine learning and human consensus predictors. While the human median-based predictor obtains slightly better headline figures in all measures, the difference between the two is small and far below statistical significance. We observe that a consensus approach among different machine learning algorithms is likely to be an improvement compared with specifying one particular algorithm in advance, unless one were very confident of which single algorithm to pick. Here, we would not have considered MLP to be our best prospect before seeing the results. A similar conclusion applies to human predictors.

Further, we have carried out a similar comparison of the best machine learning algorithm and the best performing human expert. Choosing these as the ‘best’ predictors would have required post hoc knowledge of the results. Here, even the headline result was a virtual tie between the top human and the best algorithm, and there was clearly no significant difference in predictive power.

Both these results lead to the conclusion that machine learning algorithms and human experts predict aqueous solubility essentially equally well. The machine learners had access to over a hundred descriptors for each compound, essentially infallible memory, and the ability to implement intricately designed algorithmic procedures with fast and precise numerical calculations. Thus it is perhaps surprising that they were unable to outperform humans at this task. Our experiences with this study, however, suggest that the prediction of solubility for more than around 25 molecules in one sitting would become an onerous task for most humans, whereas a computer is unlikely to complain if asked to make predictions for a thousand compounds. Thus, our experimental design was somewhat contrived to minimise the machines’ inherent advantage of an essentially unlimited attention span.

Even if a human and machine were chess players of equal strength, one might expect that they would calculate their best moves in different ways, the human’s experience and understanding versus the machine’s fast, accurate and extensive computation. One might speculate as to whether or not a similar dichotomy of approach applies here. While we did not ask participants to explain their methods in the survey itself, two experts subsequently informally reported applying a kind of nearest neighbour algorithm, looking for training set molecules similar to the query compound and then making a judgement as to whether the chemical variations between them would increase or decrease solubility. It might seem surprising that a computer could not, with the advantages described above, outperform a human at such a task. Nonetheless, human participants in the FoldIt project have been able to make useful contributions even in a field as apparently computation-intensive as protein folding, at least once the problem was suitably gamified [76]. Perhaps, some of the experts may have been confident enough in their chemist’s intuition to estimate solubilities without consciously performing an explicit computation. However, our test set was selected to contain relatively unfamiliar compounds to minimise the risk of such a task being performed simply by recall.

A minimally useful prediction has an RMSE very close to the standard deviation of sample solubilities, and can be emulated by the very simple and naïve estimation procedure of computing a mean solubility and then predicting this value for every compound. In our experiment, only around half of the humans outperformed this standard. However, nine out of ten machine learners managed this, so the overall machine learning quality is substantially better than a minimally useful predictor. Thus, we see no reason to deviate from the fairly well established view that current machine predictors are neither poor enough to fail the usefulness criterion (RMSE around 1.8 log S units in this study), nor good enough for their achievements to be limited only by the uncertainty in experimental solubility data (RMSE approximately 0.6–0.7) [9]. Machine prediction is currently somewhat better than the middle of that range, in this study at around an RMSE of 1.0 log S units. Eight machine learning algorithms and four human experts, along with both consensus predictors, appear superior to a first principles method, which obtained an RMSE of 1.45 on a similar and overlapping, though not identical, set of 25 molecules [44]. The latter approach, however, is systematically improvable and provides valuable insight by breaking solubility down to separate sublimation and hydration, and enthalpy and entropy, terms. Considering the best and consensus machine learning and human predictors, these four performed in a range of RMSE of approximately 0.95–1.15, which is numerically slightly better than the machine learning models previously described for the same overall dataset of 100 molecules [13]. However, the different experimental designs and validation strategies preclude direct quantitative comparison. Nonetheless, those individual machine learning approaches common to the two studies, RF, SVM and PLS, gave similar RMSEs to within around ± 0.1 in each case.

We observe that the consensus predictors, and the human one in particular, benefit substantially from the wisdom of crowds effect. The median-based consensus human predictor was significantly better than 13 out of the 17 individual humans with respect to its prediction errors, even for a small dataset on which statistical significance is hard to demonstrate. One might argue that this effect is masking a superiority of individual machine learning methods in at least one aspect of performance, given that nine out of ten algorithms generate useful predictions compared with around half the humans. However, there were at least two very strong predictors among the humans, competitive with any machine learning approach.

## Conclusions

We conclude that human experts can predict aqueous solubility of druglike molecules essentially equally well as machine learning algorithms. We found that the best human predictor and the best machine learning algorithm, a multi-layer perceptron, gave almost identical prediction quality. We constructed median-based consensus predictors for both human predictions and machine learning ones. While the consensus human predictor achieved very slightly better headline figures on various statistical measures, the difference between it and the consensus machine learning predictor was both small and statistically insignificant. We observe that the collection of machine learning algorithms had a higher proportion of useful predictors, nine out of ten compared with around half of the humans. Despite some weak individual human predictors, the wisdom of crowds effect inherent in the median-based consensus predictor ensured a high level of accuracy for the ensemble prediction. The best and consensus predictors give RMSEs of approximately 0.95–1.15 log S units, for both machine learning and human experts. Given the estimated uncertainty in available experimental data, the best possible predictors on existing data might achieve RMSEs around 0.6–0.7, though this figure is subject to debate, while a minimally useful predictor would be around 1.8 log S units for our dataset. Thus the current state of prediction, for both humans and machines, is somewhat better than the middle of the range between minimally useful and best realistically possible predictors.

## Additional files

**Additional file 1.** The names and structures of 75 compounds in the training set, their solubilities, and a literature source for each solubility value.

**Additional file 2.** The names and structures of the 25 compounds in the test set, and a literature source for each solubility value.

**Additional file 3.** The name, structure and solubility data in electronic form for both training and test sets, including SMILES representations of the chemical structures. This DLS-100 dataset may be reused freely with appropriate citation of our work, no further permission is required. It is also available from the University of St Andrews research portal [14] with https://doi.org/10.17630/3a3a5abc-8458-4924-8e6c-b804347605e8 and from figshare [15] with https://doi.org/10.6084/m9.figshare.5545639.

**Additional file 4.** The ethics approval letter.

**Additional file 5.** A full copy of the survey given to the human experts.

**Additional file 6.** The machine learning computed solubility predictions.

**Additional file 7.** Ten python scripts implementing the different machine learning algorithms.

**Additional file 8.** The human experts’ solubility predictions.
