# Supplementary material for: Can human experts predict solubility better than computers?
Source: J Cheminform. 2017 Dec 13;9:63. doi: 10.1186/s13321-017-0250-y (PMC5729181; doi:10.1186/s13321-017-0250-y)
Supplement: Supplementary file 1 — Additional file 1. The names and structures of 75 compounds in the training set, their solubilities, and a literature source for each solubility value. [file 13321_2017_250_MOESM1_ESM.pdf]

Solubility Panel - Training data

75 molecules and their aqueous solubilities in logarithmic units of mol/L

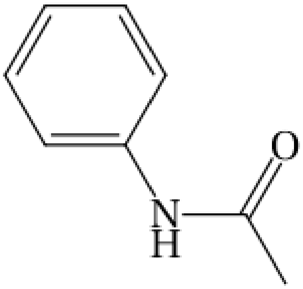

acetanilide

Rytting E, Lentz KA, Chen XQQ, Qian F, Vakatesh S. *AAPS J*, **7**:E78-E105 (2005)

-1.40

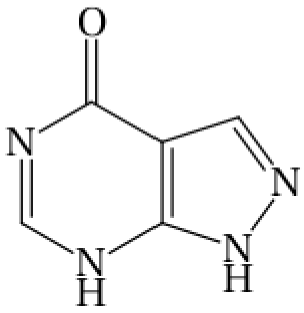

allopurinol

Bergstrom CAS, Wassvik CM, *et al*. *J Chem Inf Comput Sci*, **44**:1477−1488 (2004) **-2.26**

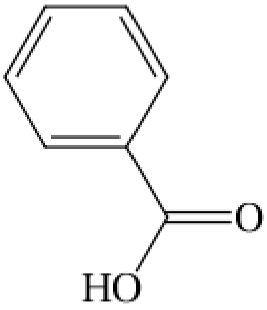

benzoic acid

Bergstrom CAS, Wassvik CM, *et al*. *J Chem Inf Comput Sci*, **44**:1477−1488 (2004) **-1.58**

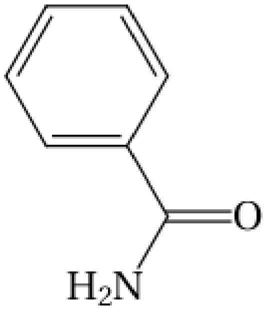

benzamide

Rytting E, Lentz KA, Chen XQQ, Qian F, Vakatesh S. *AAPS J*, **7**:E78-E105 (2005) **-0.95**

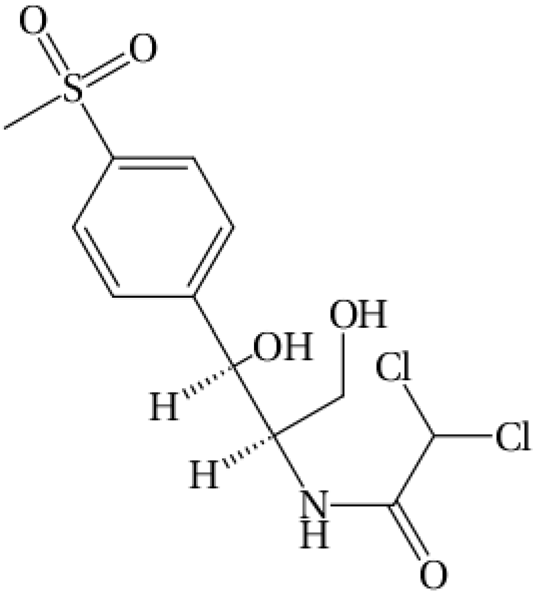

thiamphenicol

Rytting E, Lentz KA, Chen XQQ, Qian F, Vakatesh S. *AAPS J*, **7**:E78-E105 (2005) **-2.15**

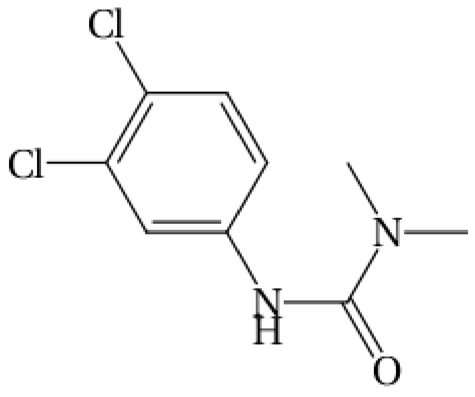

diuron

Rytting E, Lentz KA, Chen XQQ, Qian F, Vakatesh S. *AAPS J*, **7**:E78-E105 (2005) **-3.76**

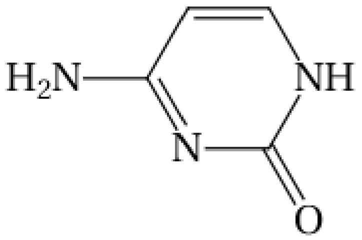

cytosine

Rytting E, Lentz KA, Chen XQQ, Qian F, Vakatesh S. *AAPS J*, **7**:E78-E105 (2005) **-1.16**

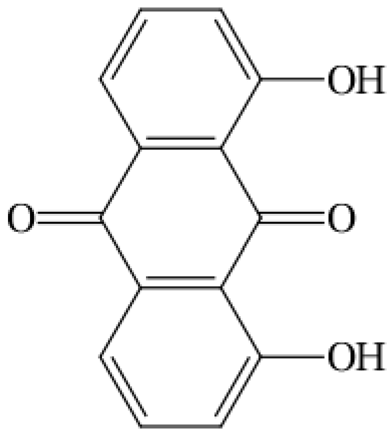

1,8-dihydroxyanthraquinone

Rytting E, Lentz KA, Chen XQQ, Qian F, Vakatesh S. *AAPS J*, **7**:E78-E105 (2005) **-5.19**

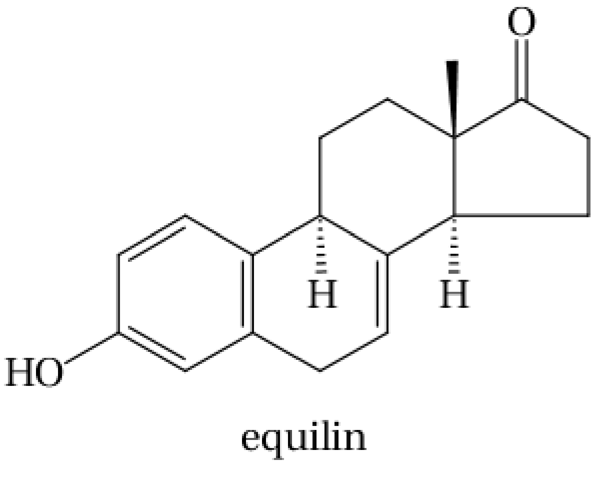

equilin

Rytting E, Lentz KA, Chen XQQ, Qian F, Vakatesh S. *AAPS J*, **7**:E78-E105 (2005) **-5.28**

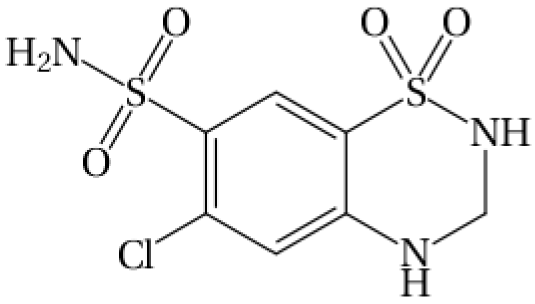

hydrochlorothiazide

Hopfinger AJ, Esposito EX, *et al*. *J Chem Inf Model*, **49**:1-5 (2008) **-2.68**

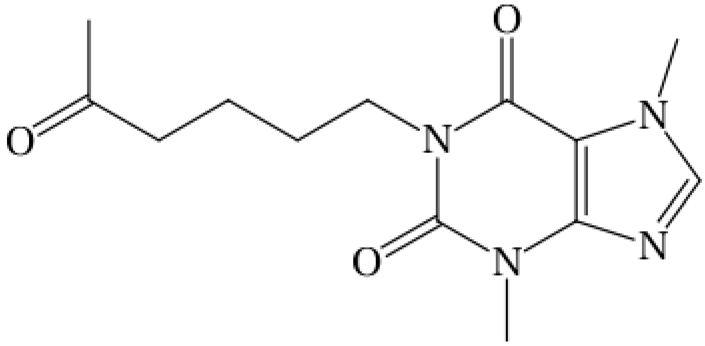

pentoxifylline

Rytting E, Lentz KA, Chen XQQ, Qian F, Vakatesh S. *AAPS J*, **7**:E78-E105 (2005) **-0.56**

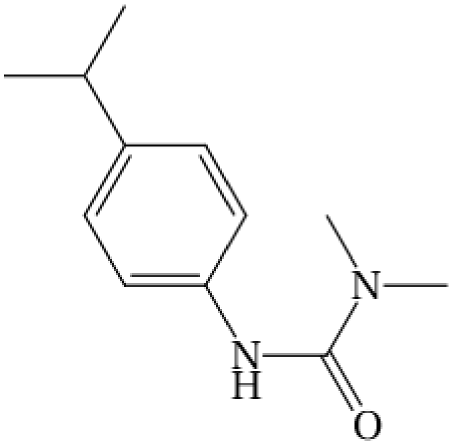

isoproturon

Rytting E, Lentz KA, Chen XQQ, Qian F, Vakatesh S. *AAPS J*, **7**:E78-E105 (2005) **-3.47**

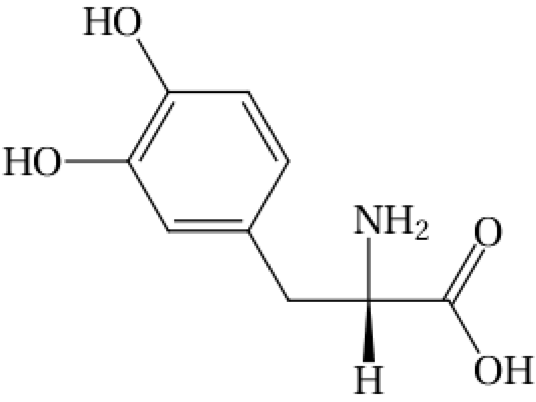

L-DOPA(levodopa)

Rytting E, Lentz KA, Chen XQQ, Qian F, Vakatesh S. *AAPS J*, **7**:E78-E105 (2005) **-1.82**

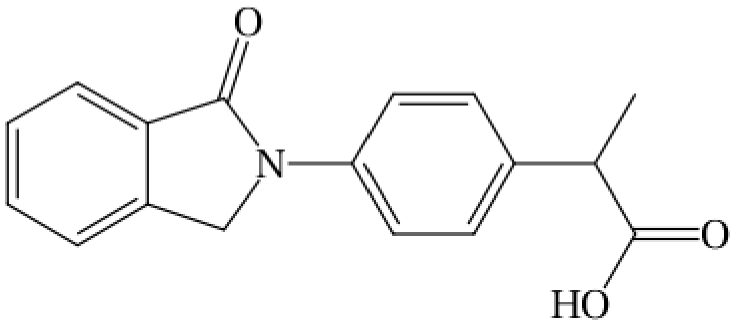

indoprofen

Bergstrom CAS, Wassvik CM, *et al*. *J Chem Inf Comput Sci*, **44**:1477−1488 (2004) **-4.82**

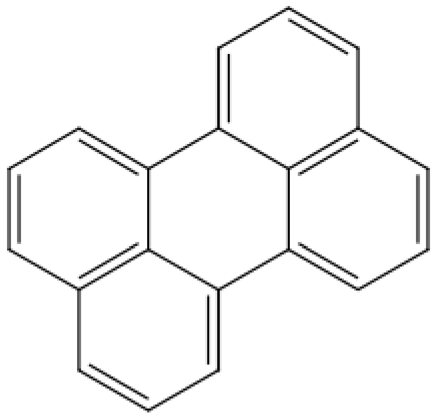

perylene

Rytting E, Lentz KA, Chen XQQ, Qian F, Vakatesh S. *AAPS J*, **7**:E78-E105 (2005) **-8.80**

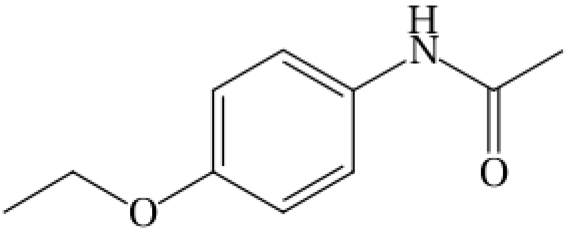

phenacetin

Rytting E, Lentz KA, Chen XQQ, Qian F, Vakatesh S. *AAPS J*, **7**:E78-E105 (2005) **-2.37**

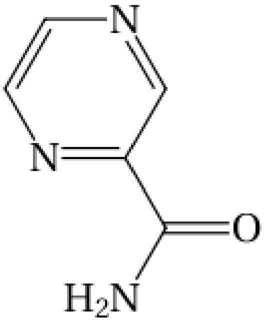

pyrazinamide

Rytting E, Lentz KA, Chen XQQ, Qian F, Vakatesh S. *AAPS J*, **7**:E78-E105 (2005) **-0.91**

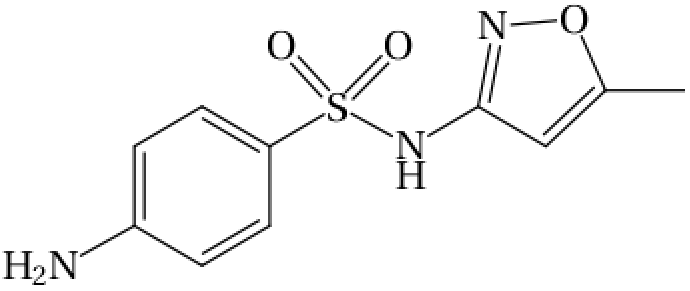

sulfamethoxazole

Rytting E, Lentz KA, Chen XQQ, Qian F, Vakatesh S. *AAPS J*, **7**:E78-E105 (2005) **-2.70**

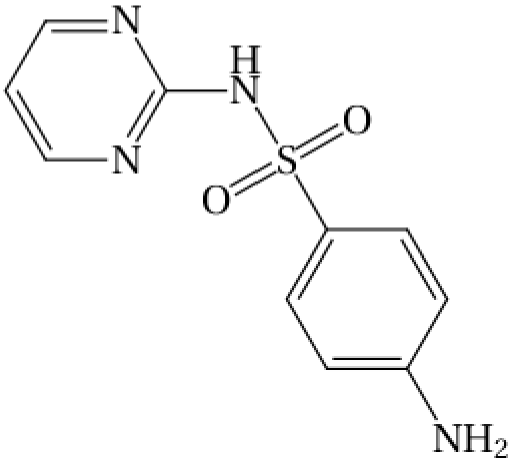

sulfadiazine

Rytting E, Lentz KA, Chen XQQ, Qian F, Vakatesh S. *AAPS J*, **7**:E78-E105 (2005) **-3.53**

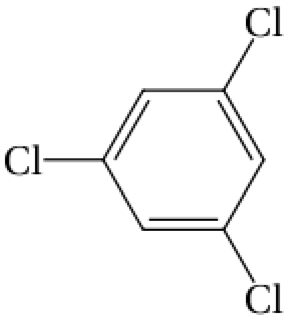

1,3,5-trichlorobenzene

Rytting E, Lentz KA, Chen XQQ, Qian F, Vakatesh S. *AAPS J*, **7**:E78-E105 (2005) **-4.44**

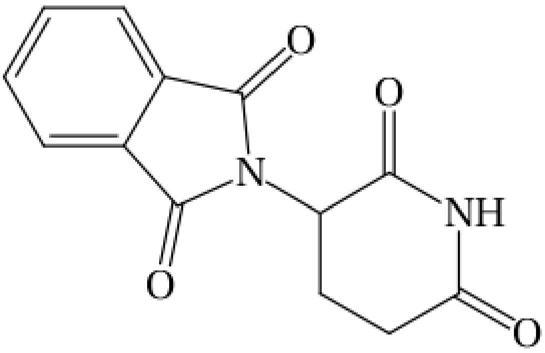

thalidomide

Rytting E, Lentz KA, Chen XQQ, Qian F, Vakatesh S. *AAPS J*, **7**:E78-E105 (2005) **-3.70**

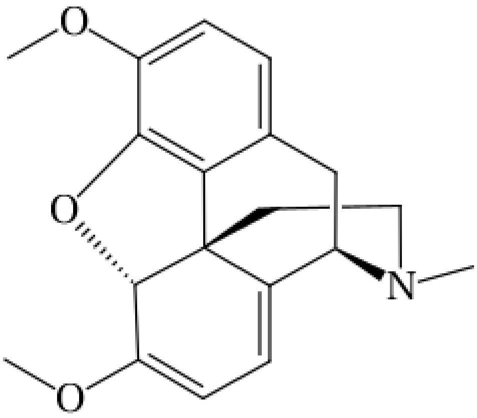

thebaine

Rytting E, Lentz KA, Chen XQQ, Qian F, Vakatesh S. *AAPS J*, **7**:E78-E105 (2005) **-2.66**

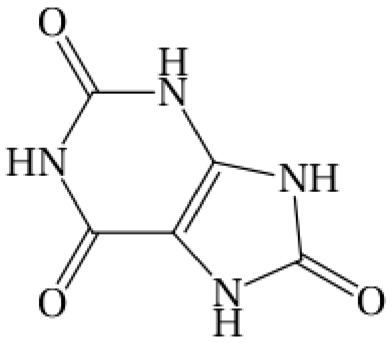

uric acid

Rytting E, Lentz KA, Chen XQQ, Qian F, Vakatesh S. *AAPS J*, **7**:E78-E105 (2005) **-3.40**

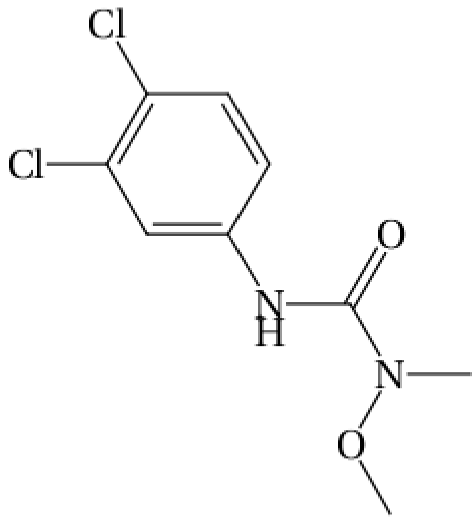

linuron

Rytting E, Lentz KA, Chen XQQ, Qian F, Vakatesh S. *AAPS J*, **7**:E78-E105 (2005) **-3.52**

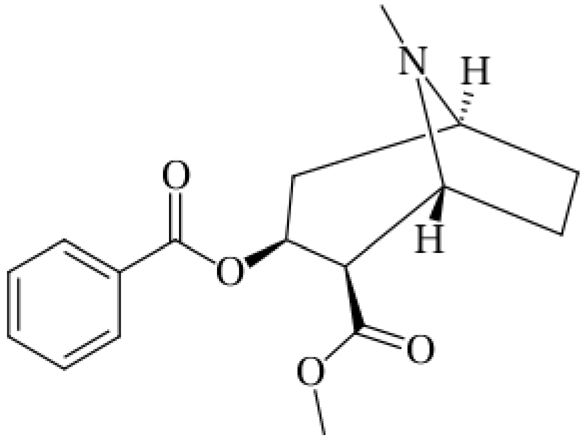

cocaine

Bergstrom CAS, Wassvik CM, *et al*. *J Chem Inf Comput Sci*, **44**:1477−1488 (2004) **-2.25**

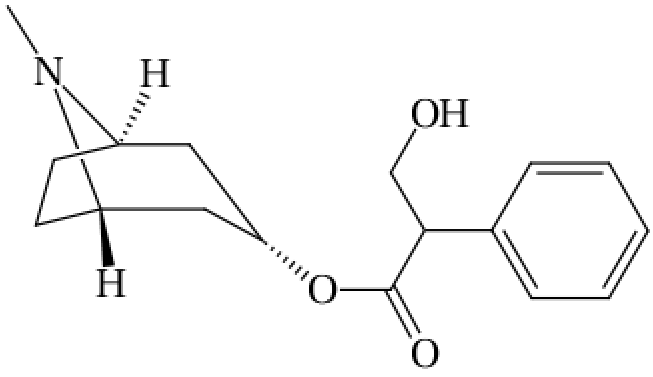

atropine

Llinas A, Glen RC, Goodman JM. *J Chem Inf Model*., **48**:1289-1303 (2008) **-2.00**

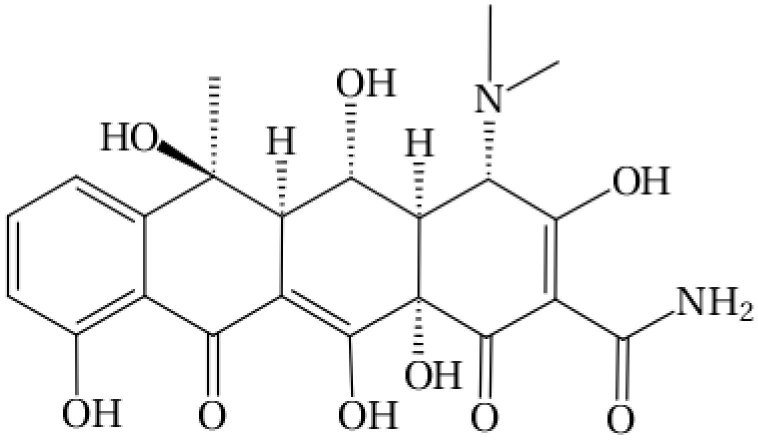

oxytetracycline

Llinas A, Glen RC, Goodman JM. *J Chem Inf Model*., **48**:1289-1303 (2008) **-3.09**

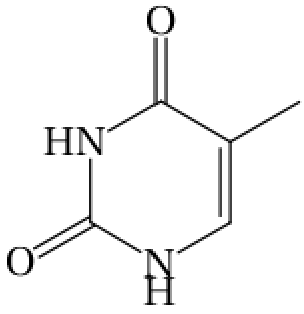

thymine

Rytting E, Lentz KA, Chen XQQ, Qian F, Vakatesh S. *AAPS J*, **7**:E78-E105 (2005) **-1.50**

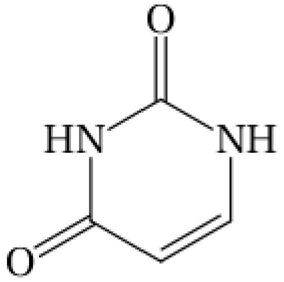

uracil

Bergstrom CAS, Wassvik CM, *et al*. *J Chem Inf Comput Sci*, **44**:1477−1488 (2004) **-1.49**

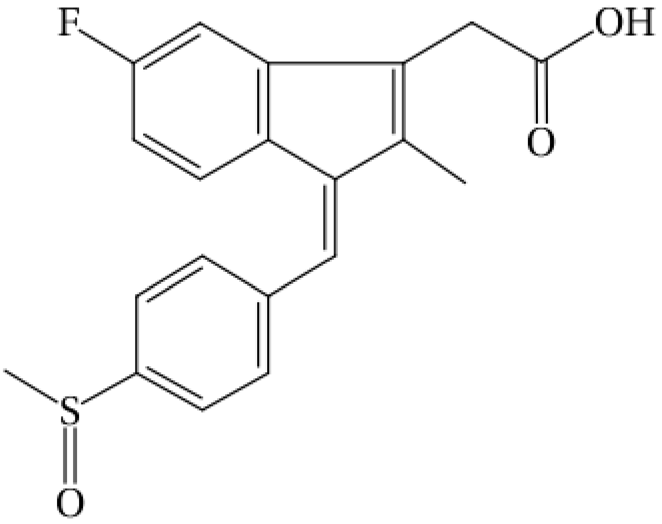

sulindac

Llinas A, Glen RC, Goodman JM. *J Chem Inf Model*., **48**:1289-1303 (2008)

-4.50

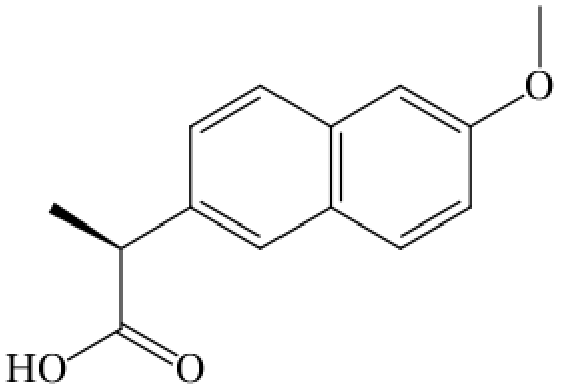

naproxen

Llinas A, Glen RC, Goodman JM. *J Chem Inf Model*., **48**:1289-1303 (2008)

-4.50

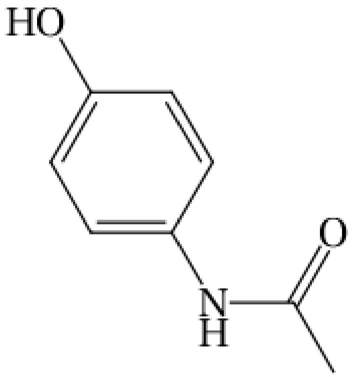

paracetamol

Llinas A, Glen RC, Goodman JM. *J Chem Inf Model*., **48**:1289-1303 (2008) **-1.06**

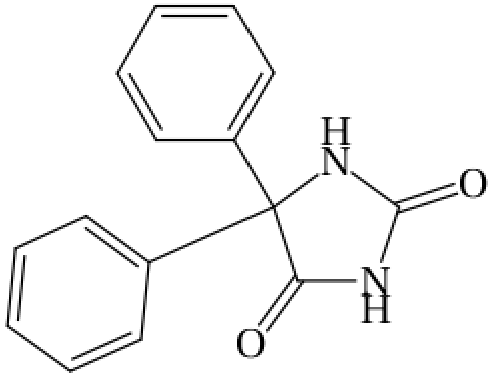

5,5-diphenylhydantoin

Llinas A, Glen RC, Goodman JM. *J Chem Inf Model*., **48**:1289-1303 (2008) **-3.86**

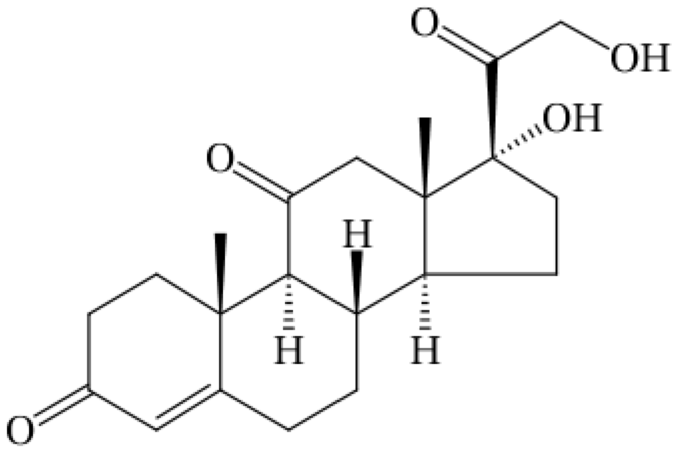

cortisone

Rytting E, Lentz KA, Chen XQQ, Qian F, Vakatesh S. *AAPS J*, **7**:E78-E105 (2005) **-3.27**

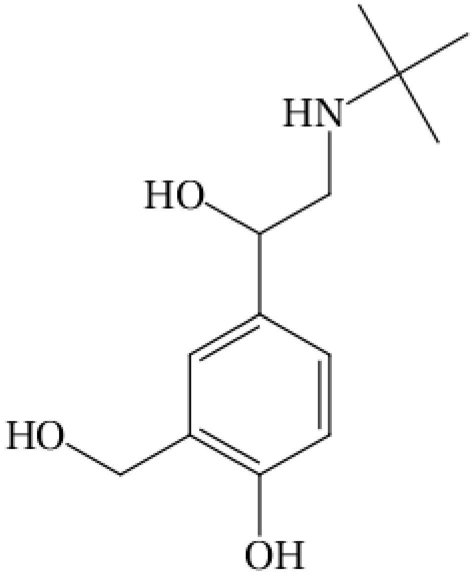

salbutamol

Rytting E, Lentz KA, Chen XQQ, Qian F, Vakatesh S. *AAPS J*, **7**:E78-E105 (2005) **-1.22**

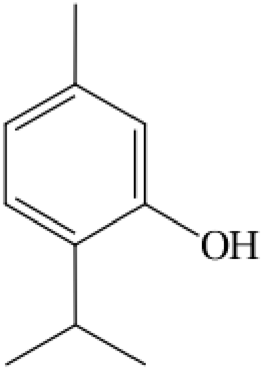

thymol

Llinas A, Glen RC, Goodman JM. *J Chem Inf Model*., **48**:1289-1303 (2008) **-2.19**

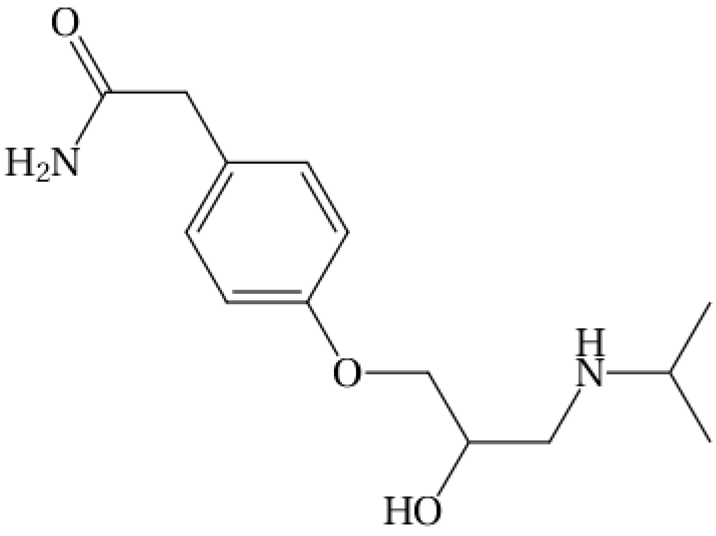

(RS)-atenolol

Narasimham LYS, Barhate VD. *J Pharmacy Res*, **4**:532-536 (2011) **-1.21**

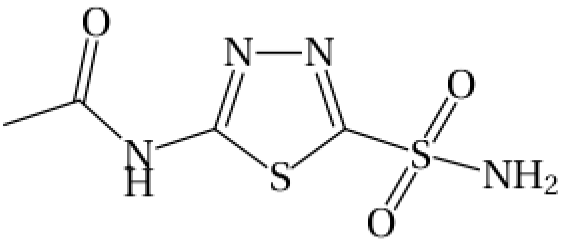

acetazolamide

Llinas A, Glen RC, Goodman JM. *J Chem Inf Model*., **48**:1289-1303 (2008) **-2.44**

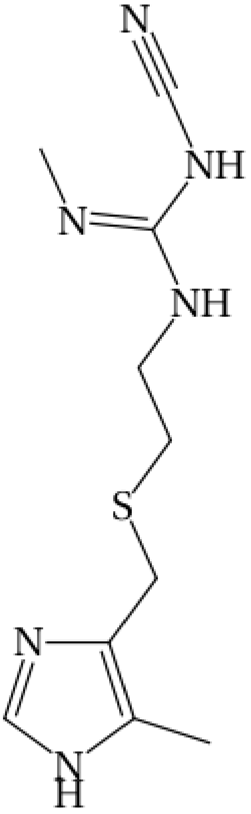

cimetidine

Llinas A, Glen RC, Goodman JM. *J Chem Inf Model*., **48**:1289-1303 (2008) **-1.69**

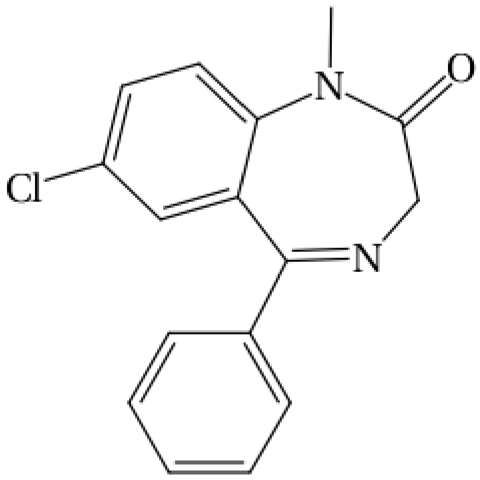

diazepam

Bergstrom CAS, Wassvik CM, *et al*. *J Chem Inf Comput Sci*, **44**:1477−1488 (2004) **-3.75**

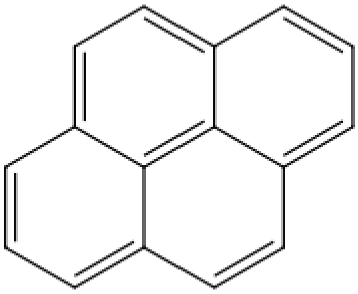

pyrene

Rytting E, Lentz KA, Chen XQQ, Qian F, Vakatesh S. *AAPS J*, **7**:E78-E105 (2005) **-6.18**

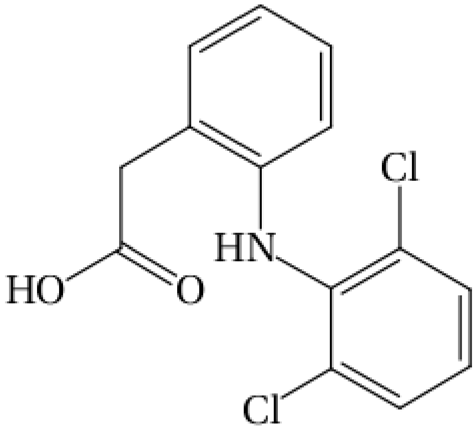

diclofenac

Llinas A, Glen RC, Goodman JM. *J Chem Inf Model*., **48**:1289-1303 (2008) **-5.46**

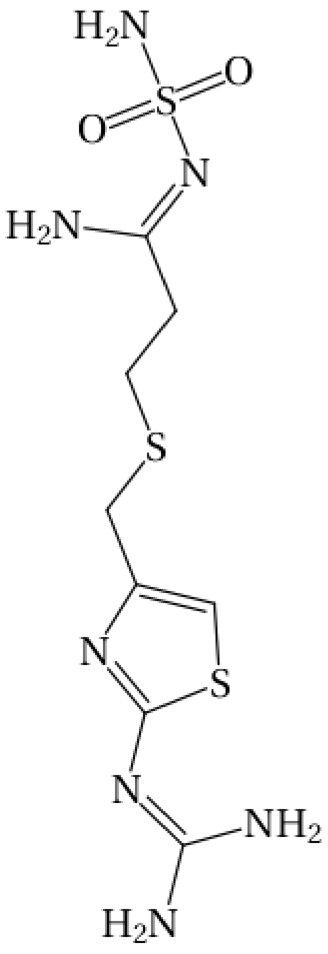

famotidine

Llinas A, Glen RC, Goodman JM. *J Chem Inf Model*., **48**:1289-1303 (2008) **-2.65**

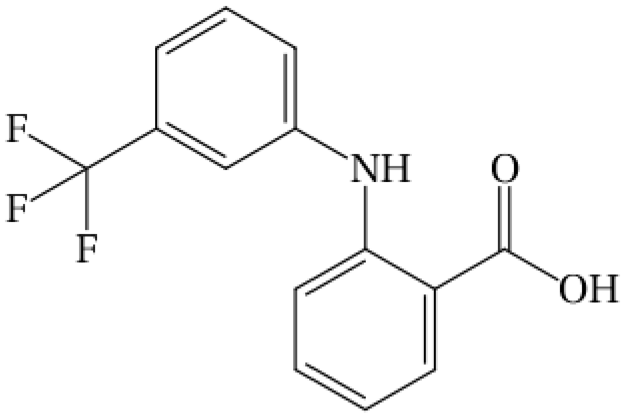

flufenamic acid

Llinas A, Glen RC, Goodman JM. *J Chem Inf Model*., **48**:1289-1303 (2008) **-5.35**

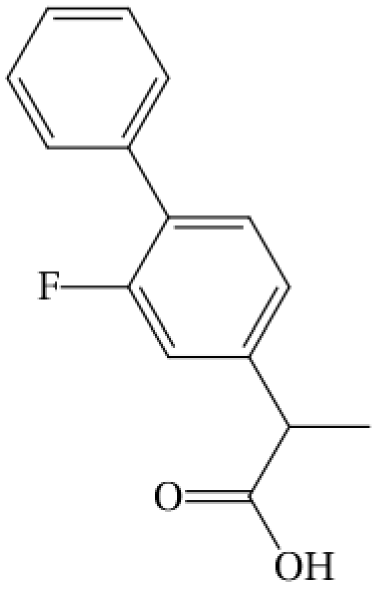

flurbiprofen

Llinas A, Glen RC, Goodman JM. *J Chem Inf Model*., **48**:1289-1303 (2008) **-4.15**

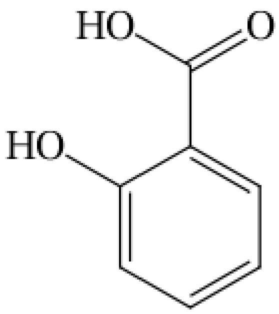

salicyclic acid

Hopfinger AJ, Esposito EX, *et al*. *J Chem Inf Model*, **49**:1-5 (2008) **-1.93**

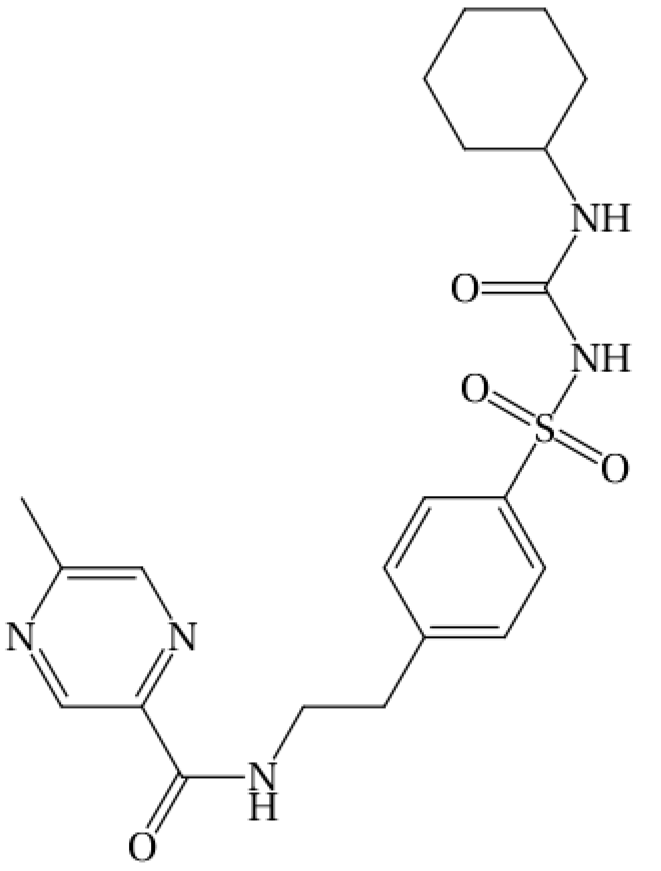

glipizide

Llinas A, Glen RC, Goodman JM. *J Chem Inf Model*., **48**:1289-1303 (2008) **-5.49**

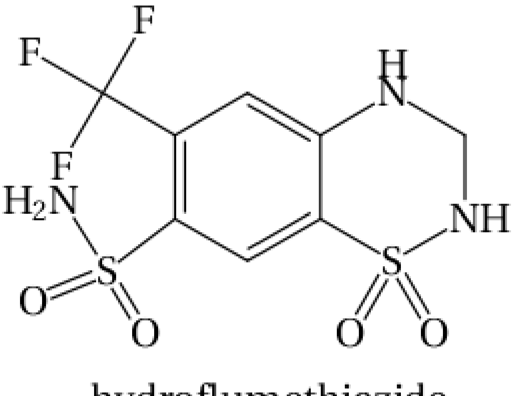

hydroflumethiazide

Llinas A, Glen RC, Goodman JM. *J Chem Inf Model*., **48**:1289-1303 (2008) **-2.97**

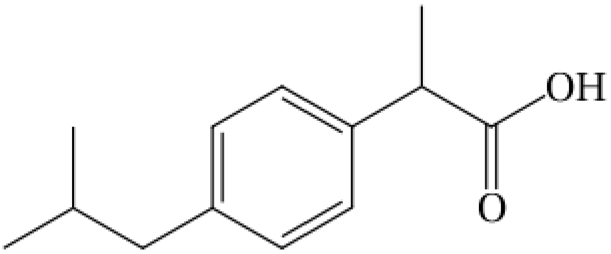

ibuprofen

Llinas A, Glen RC, Goodman JM. *J Chem Inf Model*., **48**:1289-1303 (2008) **-3.59**

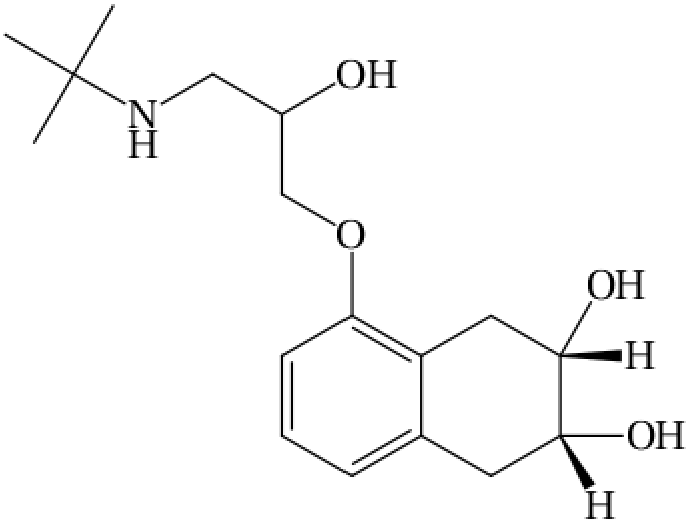

nadolol

Rytting E, Lentz KA, Chen XQQ, Qian F, Vakatesh S. *AAPS J*, **7**:E78-E105 (2005) **-1.01**

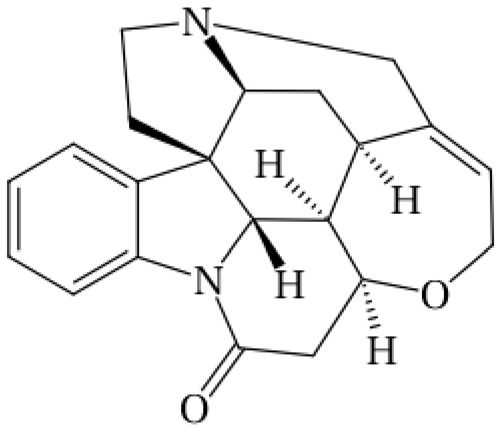

strychnine

Rytting E, Lentz KA, Chen XQQ, Qian F, Vakatesh S. *AAPS J*, **7**:E78-E105 (2005) **-3.33**

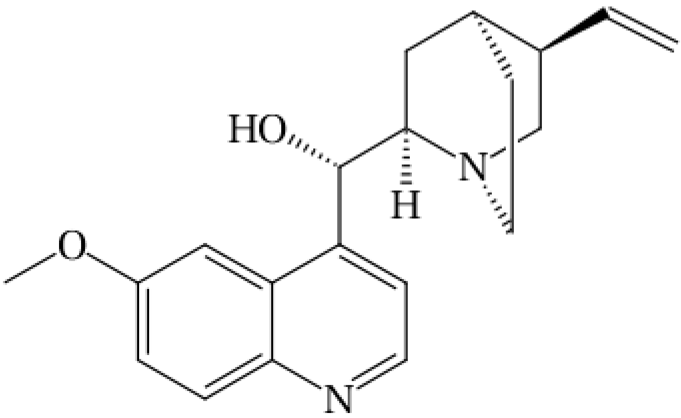

quinidine

Rytting E, Lentz KA, Chen XQQ, Qian F, Vakatesh S. *AAPS J*, **7**:E78-E105 (2005) **-2.81**

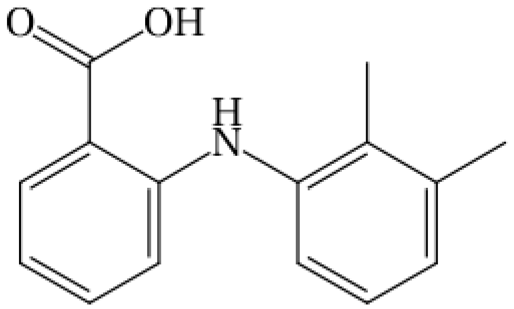

mefenamic acid

Llinas A, Glen RC, Goodman JM. *J Chem Inf Model*., **48**:1289-1303 (2008) **-6.74**

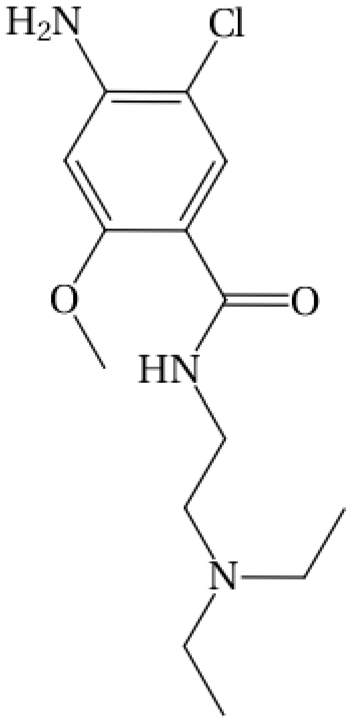

metoclopramide

Llinas A, Glen RC, Goodman JM. *J Chem Inf Model*., **48**:1289-1303 (2008) **-3.57**

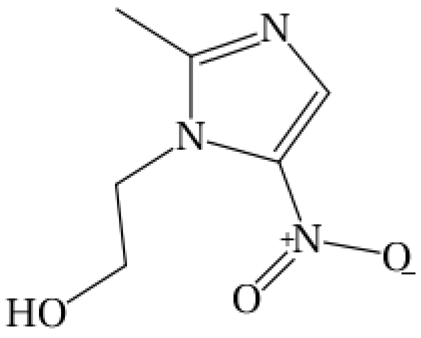

metronidazole

Llinas A, Glen RC, Goodman JM. *J Chem Inf Model*., **48**:1289-1303 (2008) **-1.22**

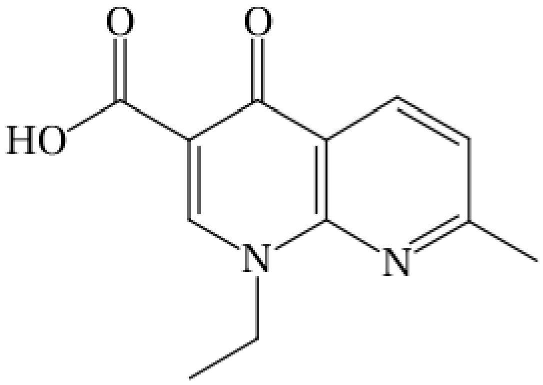

nalidixic acid

Llinas A, Glen RC, Goodman JM. *J Chem Inf Model*., **48**:1289-1303 (2008) **-3.61**

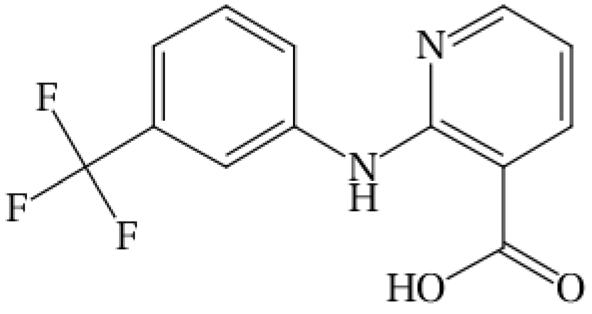

niflumic acid

Llinas A, Glen RC, Goodman JM. *J Chem Inf Model*., **48**:1289-1303 (2008) **-4.58**

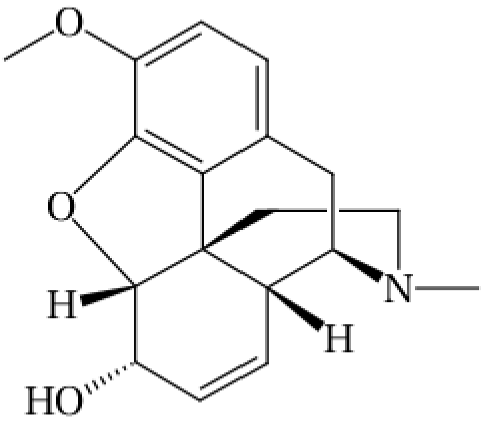

codeine

Ran Y, Yalkowsky SH. *J Chem Inf Comput Sci*, **41**:354−357 (2001) **-1.52**

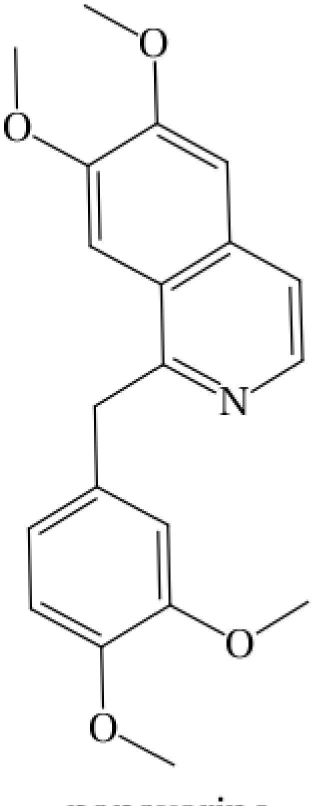

papaverine

Llinas A, Glen RC, Goodman JM. *J Chem Inf Model*., **48**:1289-1303 (2008) **-3.87**

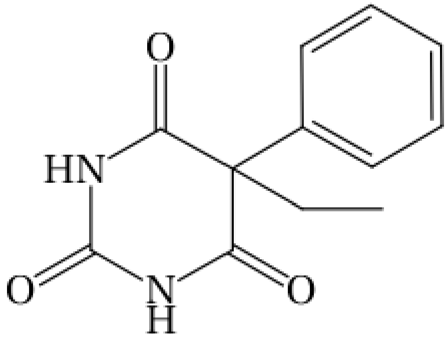

phenobarbital

Llinas A, Glen RC, Goodman JM. *J Chem Inf Model*., **48**:1289-1303 (2008)

-2.29

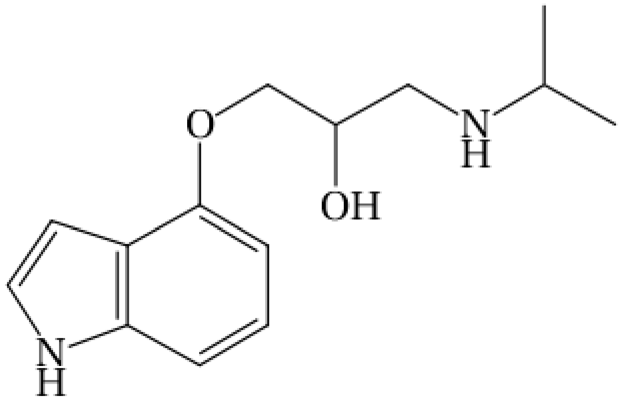

pindolol

Llinas A, Glen RC, Goodman JM. *J Chem Inf Model*., **48**:1289-1303 (2008)

-3.79

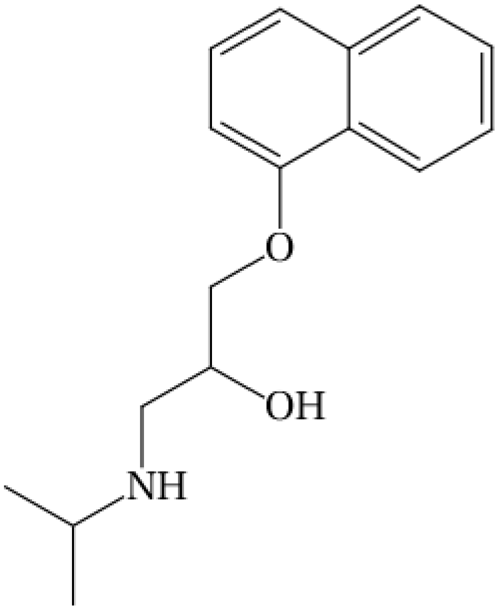

propranolol

Llinas A, Glen RC, Goodman JM. *J Chem Inf Model*., **48**:1289-1303 (2008) **-3.49**

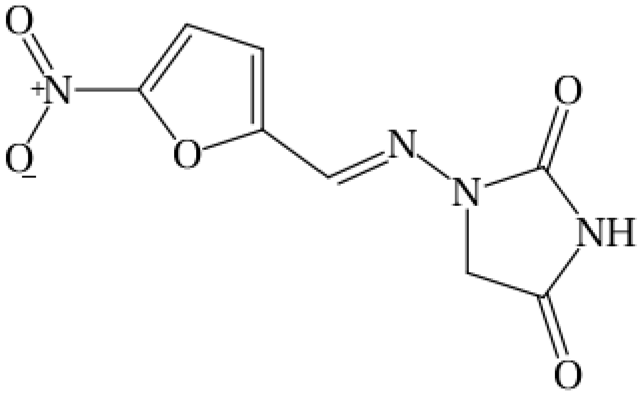

nitrofurantoin

Llinas A, Glen RC, Goodman JM. *J Chem Inf Model*., **48**:1289-1303 (2008) **-3.24**

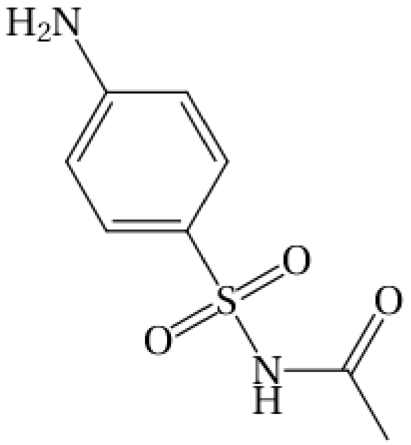

sulfacetamide

Llinas A, Glen RC, Goodman JM. *J Chem Inf Model*., **48**:1289-1303 (2008) **-1.52**

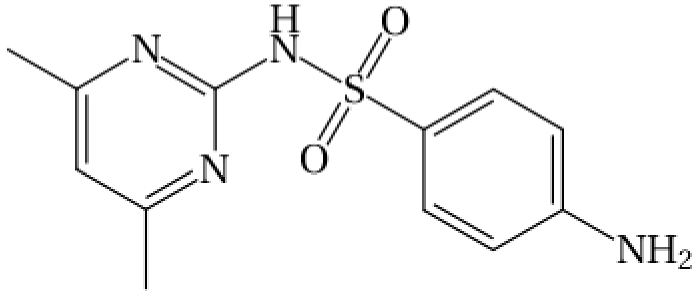

sulfamethazine

Llinas A, Glen RC, Goodman JM. *J Chem Inf Model*., **48**:1289-1303 (2008) **-2.73**

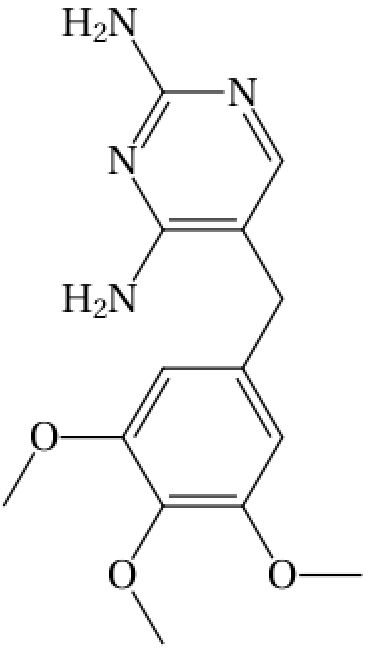

trimethoprim

Llinas A, Glen RC, Goodman JM. *J Chem Inf Model*., **48**:1289-1303 (2008) **-2.95**

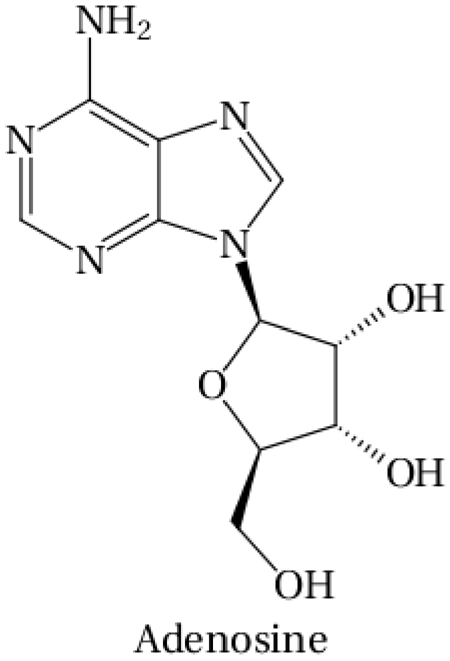

Adenosine

Rytting E, Lentz KA, Chen XQQ, Qian F, Vakatesh S. *AAPS J*, **7**:E78-E105 (2005) **-1.73**

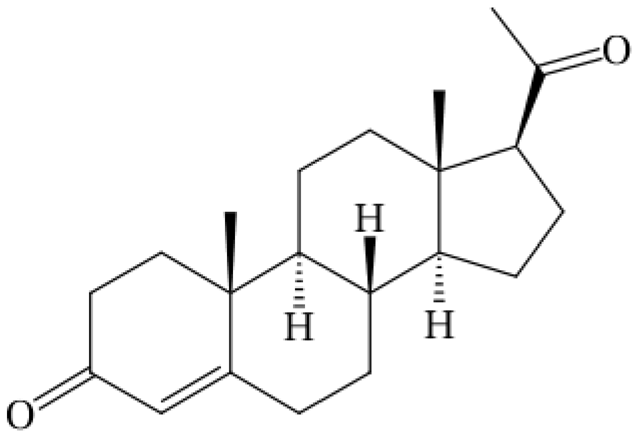

progesterone

Rytting E, Lentz KA, Chen XQQ, Qian F, Vakatesh S. *AAPS J*, **7**:E78-E105 (2005) **-4.42**

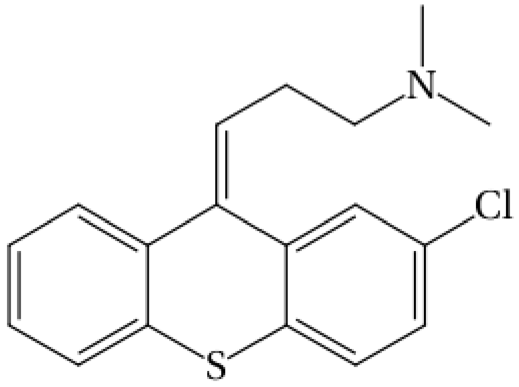

chloroprothixene

Llinas A, Glen RC, Goodman JM. *J Chem Inf Model*., **48**:1289-1303 (2008) **-6.75**

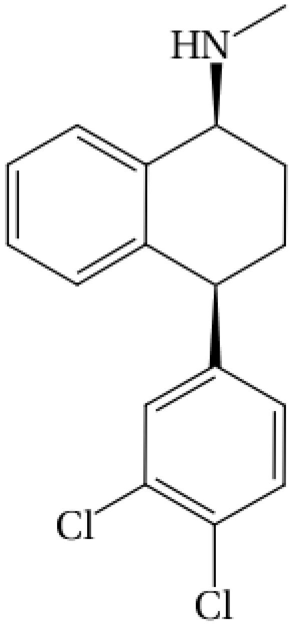

sertraline

Llinas A, Glen RC, Goodman JM. *J Chem Inf Model*., **48**:1289-1303 (2008) **-4.83**

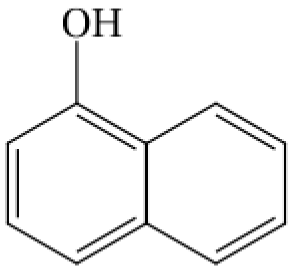

1-naphthol

Llinas A, Glen RC, Goodman JM. *J Chem Inf Model*., **48**:1289-1303 (2008) **-1.98**

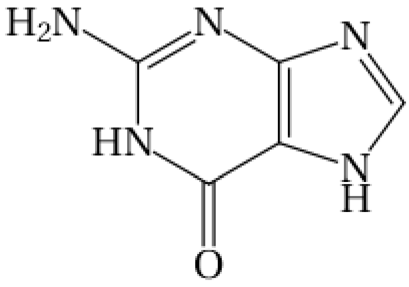

guanine

Llinas A, Glen RC, Goodman JM. *J Chem Inf Model.*, **48**:1289-1303 (2008) **-4.43**

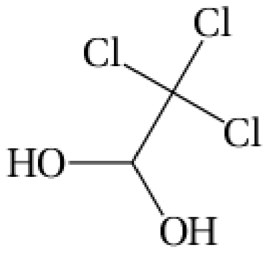

chloral hydrate

Rytting E, Lentz KA, Chen XQQ, Qian F, Vakatesh S. *AAPS J*, **7**:E78-E105 (2005) **+1.70**

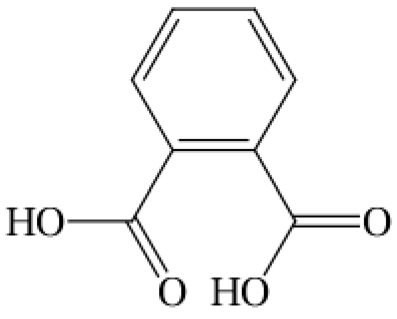

phthalic acid

Llinas A, Glen RC, Goodman JM. *J Chem Inf Model.*, **48**:1289-1303 (2008) **-1.49**

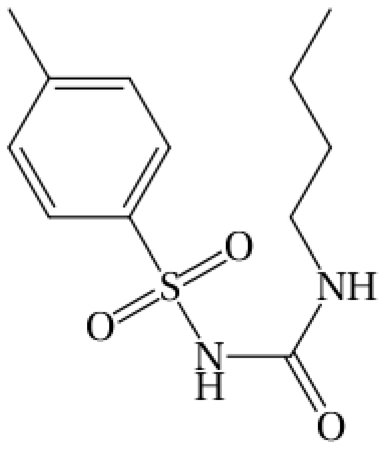

tolbutamide

Hopfinger AJ, Esposito EX, *et al*. *J Chem Inf Model*, **49**:1-5 (2008) **-3.46**
