# Supplementary material for: Can human experts predict solubility better than computers?
Source: J Cheminform. 2017 Dec 13;9:63. doi: 10.1186/s13321-017-0250-y (PMC5729181; doi:10.1186/s13321-017-0250-y)
Supplement: Supplementary file 2 — Additional file 2. The names and structures of the 25 compounds in the test set, and a literature source for each solubility value. [file 13321_2017_250_MOESM2_ESM.pdf]

Solubility Panel - Test data

25 molecules and their aqueous solubilities in logarithmic units of mol/L

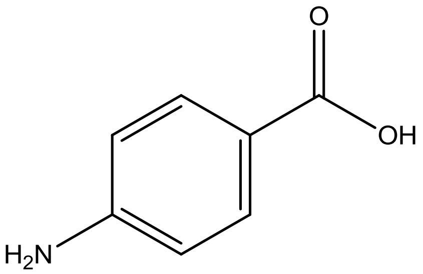

4-aminobenzoic acid

Rytting E, Lentz KA, Chen XQQ, Qian F, Vakatesh S. *AAPS J*, **7**:E78-E105 (2005) **-1.37**

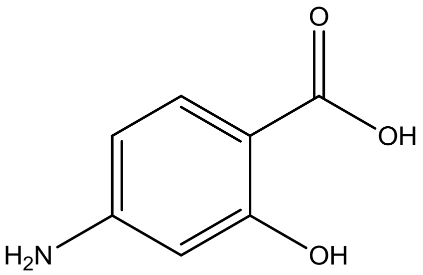

4-aminosalicylic acid

Rytting E, Lentz KA, Chen XQQ, Qian F, Vakatesh S. *AAPS J*, **7**:E78-E105 (2005) **-1.96**

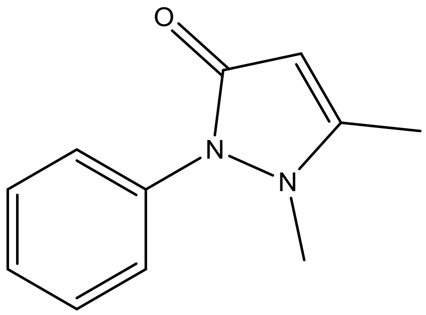

antipyrine

Rytting E, Lentz KA, Chen XQQ, Qian F, Vakatesh S. *AAPS J*, **7**:E78-E105 (2005)

+0.48

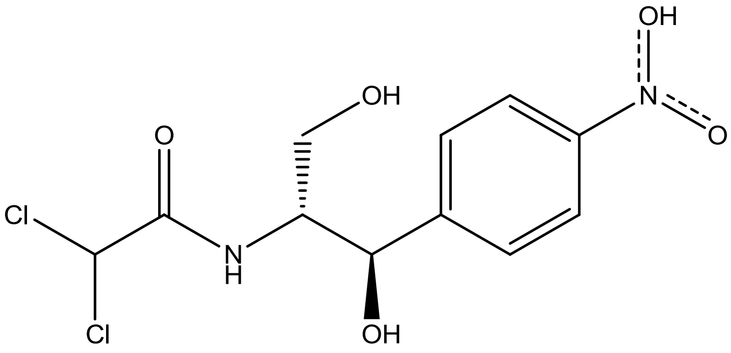

chloramphenicol

Rytting E, Lentz KA, Chen XQQ, Qian F, Vakatesh S. *AAPS J*, **7**:E78-E105 (2005)

-2.11

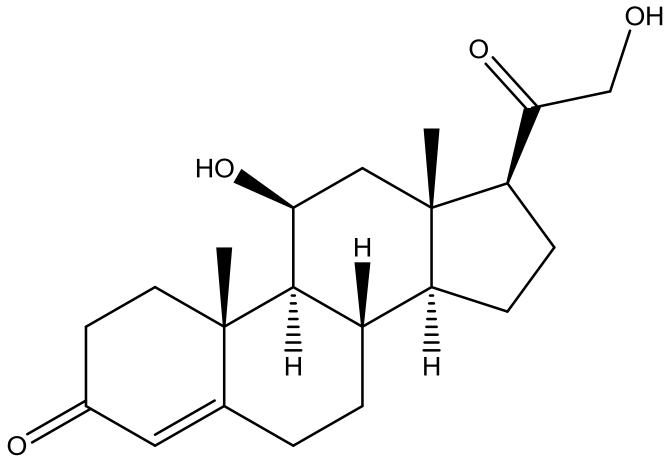

corticosterone

Bergstrom CAS, Wassvik CM, *et al*. *J Chem Inf Comput Sci*, **44**:1477−1488 (2004) **-3.24**

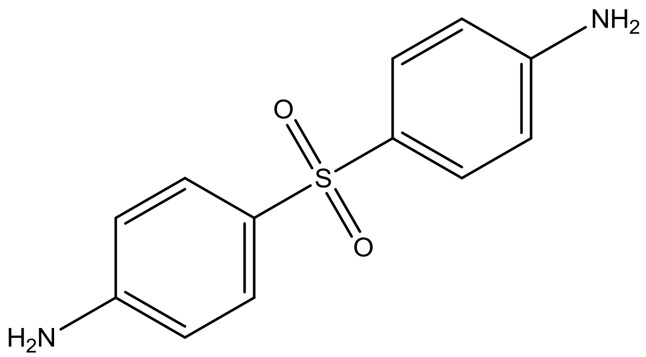

dapsone

Rytting E, Lentz KA, Chen XQQ, Qian F, Vakatesh S. *AAPS J*, **7**:E78-E105 (2005) **-3.09**

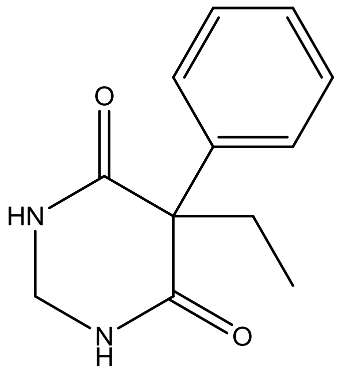

primidone

Rytting E, Lentz KA, Chen XQQ, Qian F, Vakatesh S. *AAPS J*, **7**:E78-E105 (2005) **-2.64**

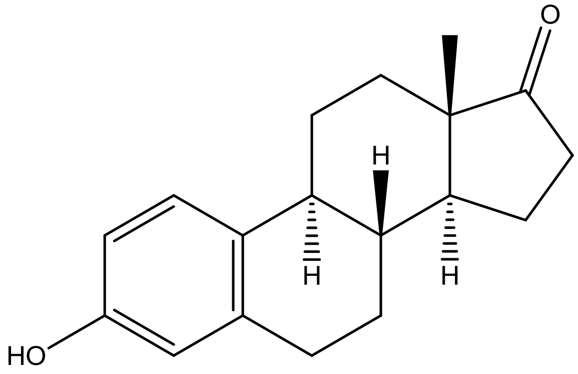

estrone

Shareef A, Angove MJ, Wells JD, Johnson BB. J Chem Eng Data, 51:879−881 (2006) **-5.32**

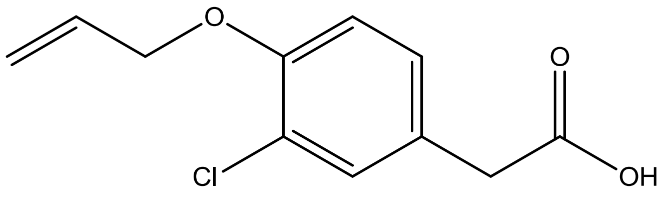

alclofenac

Bergstrom CAS, Wassvik CM, *et al*. *J Chem Inf Comput Sci*, **44**:1477−1488 (2004) **-3.13**

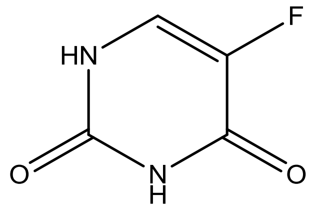

5-fluorouracil

Rytting E, Lentz KA, Chen XQQ, Qian F, Vakatesh S. *AAPS J*, **7**:E78-E105 (2005) **-1.03**

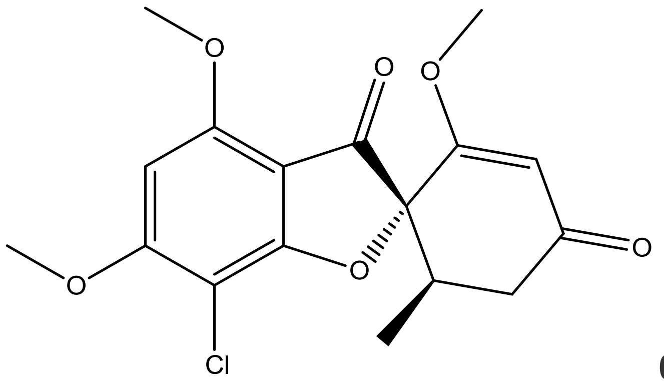

griseofulvin

Rytting E, Lentz KA, Chen XQQ, Qian F, Vakatesh S. *AAPS J*, **7**:E78-E105 (2005) **-3.25**

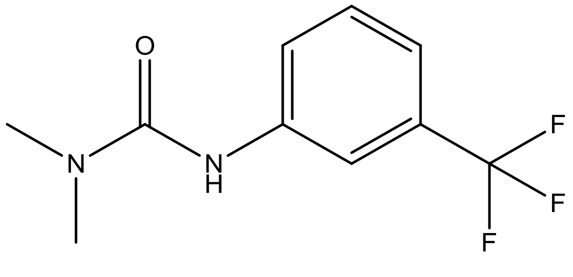

fluometuron

Rytting E, Lentz KA, Chen XQQ, Qian F, Vakatesh S. *AAPS J*, **7**:E78-E105 (2005) **-3.46**

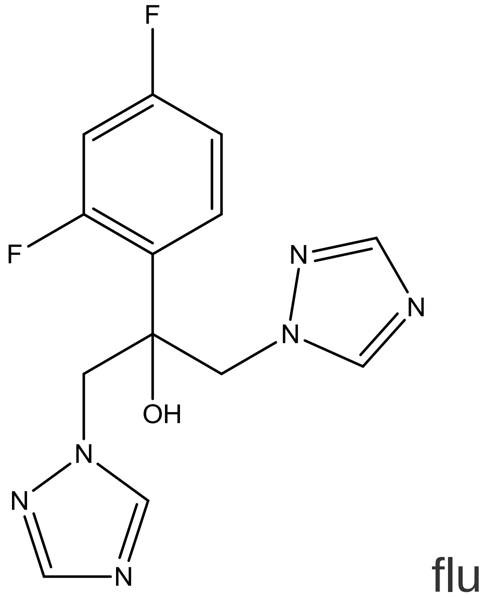

fluconazole

Bergstrom CAS, Wassvik CM, *et al*. *J Chem Inf Comput Sci*, **44**:1477−1488 (2004) **-1.80**

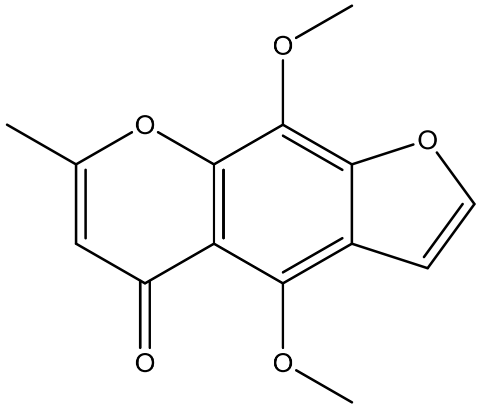

khellin

Rytting E, Lentz KA, Chen XQQ, Qian F, Vakatesh S. *AAPS J*, **7**:E78-E105 (2005) **-3.02**

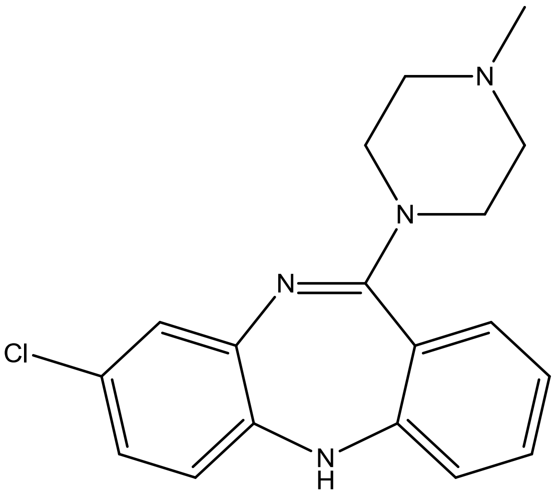

clozapine

Hopfinger AJ, Esposito EX, *et al*. *J Chem Inf Model*, **49**:1-5 (2008)

–3.24

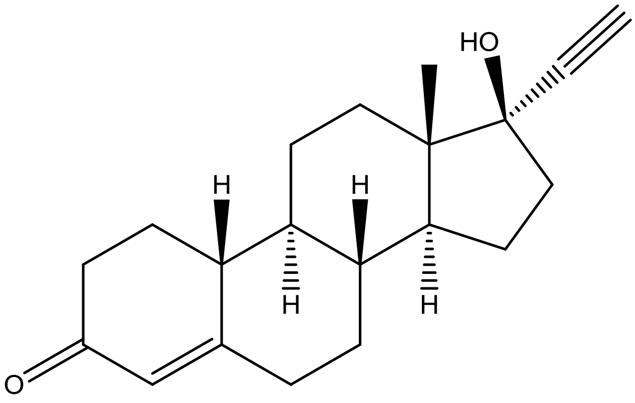

norethisterone

Rytting E, Lentz KA, Chen XQQ, Qian F, Vakatesh S. *AAPS J*, **7**:E78-E105 (2005)

-4.63

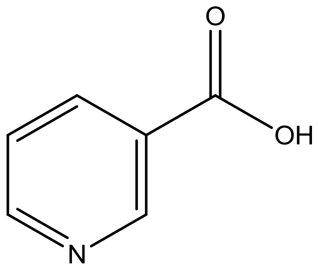

nicotinic acid

Rytting E, Lentz KA, Chen XQQ, Qian F, Vakatesh S. *AAPS J*, **7**:E78-E105 (2005) **-0.85**

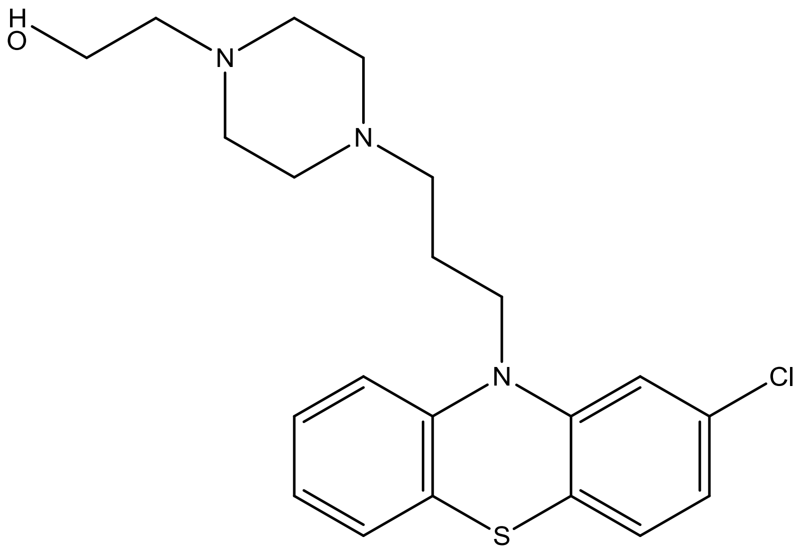

perphenazine

Rytting E, Lentz KA, Chen XQQ, Qian F, Vakatesh S. *AAPS J*, **7**:E78-E105 (2005) **-4.16**

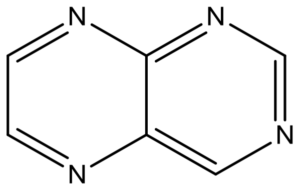

pteridine

Palmer DS, Llinas A, Morao I, Day GM, *et al*. *Mol Pharmaceutics*, **5**:266-279 (2008) **+0.02**

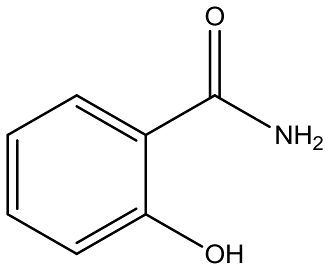

salicylamide

Rytting E, Lentz KA, Chen XQQ, Qian F, Vakatesh S. *AAPS J*, **7**:E78-E105 (2005) **-1.84**

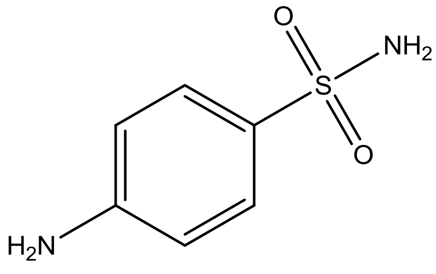

sulfanilamide

Rytting E, Lentz KA, Chen XQQ, Qian F, Vakatesh S. *AAPS J*, **7**:E78-E105 (2005) **-1.36**

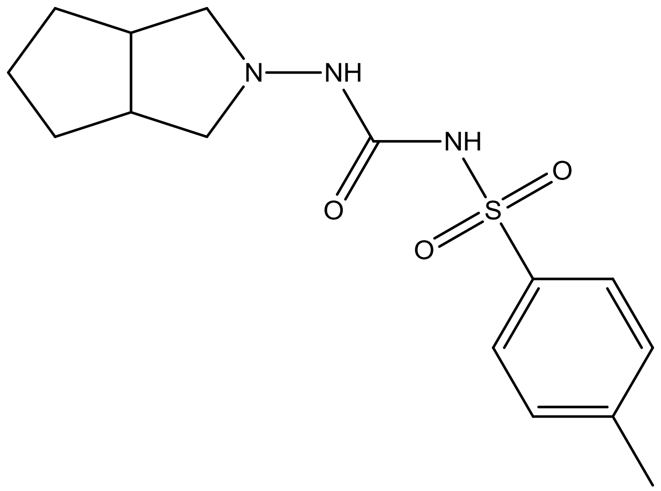

gliclazide

Narasimham LYS, Barhate VD. *J Pharmacy Res*, **4**:532-536 (2011) **-4.29**

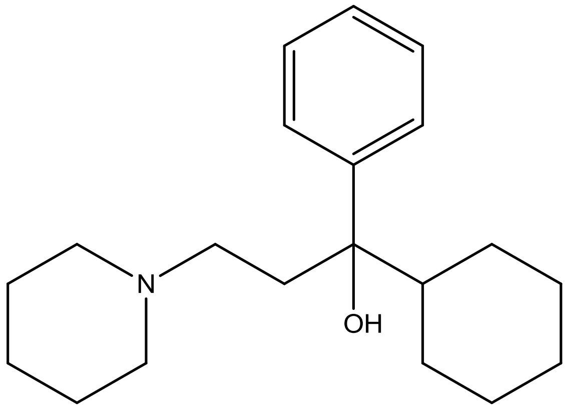

trihexyphenidyl

Bergstrom CAS, Luthman K, Artursson P. *Eur J Pharm Sci*, **22**:387–398 (2004) **-5.20**

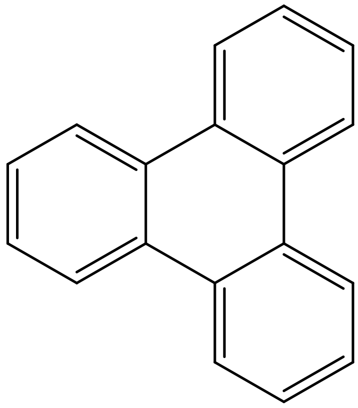

triphenylene

Rytting E, Lentz KA, Chen XQQ, Qian F, Vakatesh S. *AAPS J*, **7**:E78-E105 (2005) **-6.73**

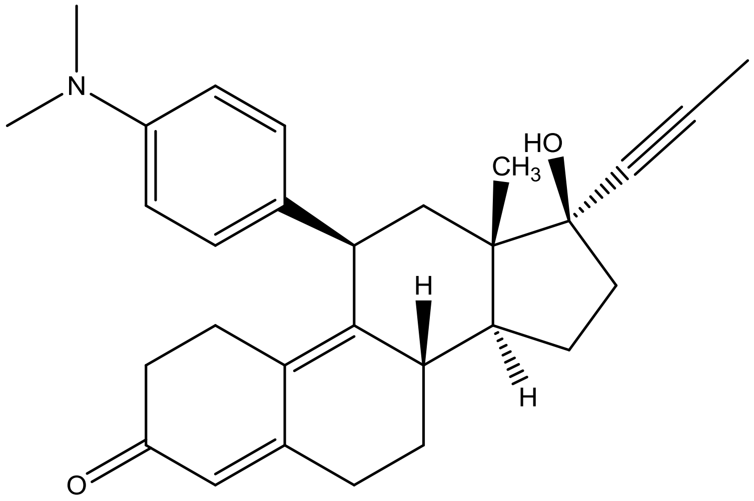

mifepristone

Bergstrom CAS, Luthman K, Artursson P. *Eur J Pharm Sci*, **22**:387–398 (2004) **-5.90**
