# Supplementary material for: Can human experts predict solubility better than computers?
Source: J Cheminform. 2017 Dec 13;9:63. doi: 10.1186/s13321-017-0250-y (PMC5729181; doi:10.1186/s13321-017-0250-y)
Supplement: Supplementary file 4 — Additional file 4. The ethics approval letter. [file 13321_2017_250_MOESM4_ESM.pdf]

University of St Andrews | FOUNDED 1413

University Teaching and Research Ethics Committee

12 October 2016

Dear Samuel and John

Thank you for submitting your ethical application which was considered at the School of Psychology & Neuroscience Ethics Committee meeting on 6th October 2016; the following documents have been reviewed:

1. Ethical Application Form
2. Project synopsis
3. Email to potential participants
4. Participant Information Sheet
5. Consent Form
6. Debriefing Form
7. Questionnaire
8. Data Management Plan

The School of Psychology & Neuroscience Ethics Committee has been delegated to act on behalf of the University Teaching and Research Ethics Committee (UTREC) and has granted this application ethical approval. The particulars relating to the approved project are as follows -

**Approval Code:**	PS12383	**Approved on:**	11/10/2016	**Approval Expiry:**	11/10/2021
**Project Title:**	Can human experts predict solubility better than computers?				
**Researchers:**	Samuel Boobier and Dr John Mitchell				
**Supervisor:**	Dr John Mitchell				

Approval is awarded for five years. Projects which have not commenced within two years of approval must be re-submitted for review by your School Ethics Committee. If you are unable to complete your research within the five year approval period, you are required to write to your School Ethics Committee Convener to request a discretionary extension of no greater than 6 months or to re-apply if directed to do so, and you should inform your School Ethics Committee when your project reaches completion.

If you make any changes to the project outlined in your approved ethical application form, you should inform your supervisor and seek advice on the ethical implications of those changes from the School Ethics Convener who may advise you to complete and submit an ethical amendment form for review.

Any adverse incident which occurs during the course of conducting your research must be reported immediately to the School Ethics Committee who will advise you on the appropriate action to be taken.

Approval is given on the understanding that you conduct your research as outlined in your application and in compliance with UTREC Guidelines and Policies (http://www.st-andrews.ac.uk/utrec/guidelinespolicies/). You are also advised to ensure that you procure and handle your research data within the provisions of the Data Provision Act 1998 and in accordance with any conditions of funding incumbent upon you.

Yours sincerely

M P Latimer

Convener of the School Ethics Committee

School of Psychology & Neuroscience, St Mary's Quad, South Street, St Andrews, Fife KY16 9JP
Email: psyethics@st-andrews.ac.uk Tel: 01334 462071

The University of St Andrews is a charity registered in Scotland: No SC013532
