# Supplementary material for: Can human experts predict solubility better than computers?
Source: J Cheminform. 2017 Dec 13;9:63. doi: 10.1186/s13321-017-0250-y (PMC5729181; doi:10.1186/s13321-017-0250-y)
Supplement: Supplementary file 5 — Additional file 5. A full copy of the survey given to the human experts. [file 13321_2017_250_MOESM5_ESM.pdf]

Participant Information

Thank you for choosing to participate in this Solubility panel.

We are examining the hypothesis that human experts are as good as computation in predicting aqueous solubility of organic molecules.

You will be asked for information about your level of formal education and your current (or most recent) field of employment, work or study.

You will then be asked to predict the aqueous solubility (in logarithmic units referred to moles per litre) of 50 druglike organic molecules.

We will endeavour to use compounds whose solubilities are experimentally known, but are not so common or significant as to be widely disseminated. However, we may throw in some better known compounds to test how this affects the results. If you believe that you know the solubility of a compound accurately, please enter this value as your prediction anyhow.

Please do not use reference books, papers or the internet to look up solubility values while taking the survey. You can choose not to make an estimate for a compound if you so wish. You may withdraw from the survey at any time.

After data collection, the raw data will be analysed and results entered into a spreadsheet. Since the data are anonymous, we do not anticipate any significant risk to the respondents' anonymity or security. Data will be securely stored on these computers indefinitely. In the event of publication, anonymous data from the study will made available on the University's Research Repository, Pure, as data supporting the relevant paper. Data will be processed using computers within the University.

At the end of the survey you will be shown a page of debriefing information.

Consent Form

This research has been scrutinised and been granted Ethical Approval through the University of St Andrews ethical approval process.

All your responses to this survey will be anonymous. Data will be handled securely and you will not be identifiable by your responses.

Once again you may choose to leave any question blank and may leave the survey at any point.

By continuing you are consenting for your responses to be used in our research.

Instructions

You will be shown 25 drug-like molecules.

Your task is to predict the solubility, in logarithmic units of mol/L, of each molecule.

To help you we have compiled a set of training data.

The training data consists of 75 molecules and their solubilities.

Click here to launch the training data in a new tab.

Qualification and Job

What is the highest level of qualification you have gained?

- A-Level/Scottish Highers (School leavers level)
- First undergraduate degree (e.g. BSc/MChem)
- PhD/Doctorate
- Other

What is your current (or most recent) occupation?

- Undergraduate Student
- Postgraduate Student
- Research academic
- Pharmaceutical industry
- Other

solubility question 1

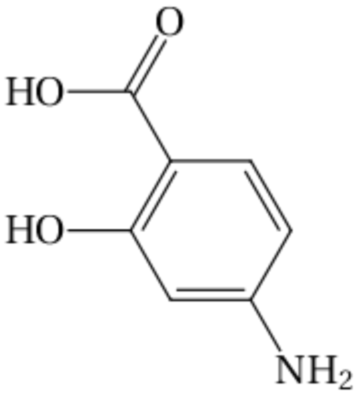

4-aminosalicylic acid

Refer to the training data.
What is the aqueous solubility, in logarithmic units of mol/L, of this molecule?

solubility question 2

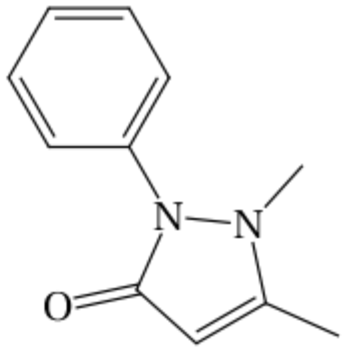

antipyrine

Refer to the training data.
What is the aqueous solubility, in logarithmic units of mol/L, of this molecule?

solubility question 3

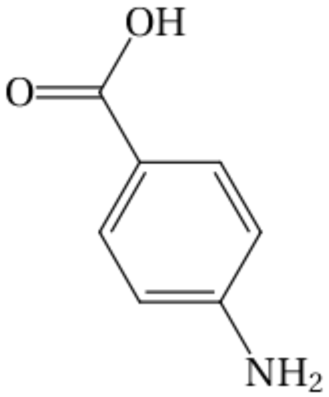

4-aminobenzoic acid

Refer to the training data.
What is the aqueous solubility, in logarithmic units of mol/L, of this molecule?

solubility question 4

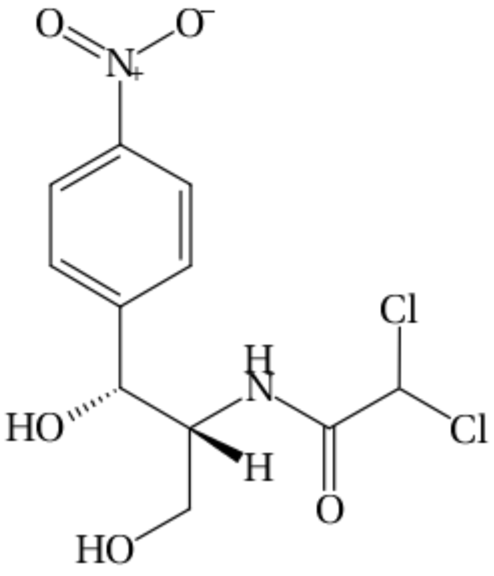

chloramphenicol

Refer to the training data.
What is the aqueous solubility, in logarithmic units of mol/L, of this molecule?

solubility question 5

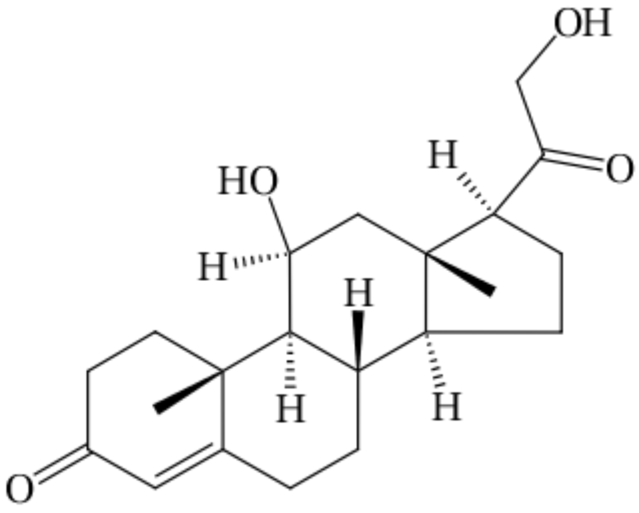

corticosterone

Refer to the training data.
What is the aqueous solubility, in logarithmic units of mol/L, of this molecule?

solubility question 6

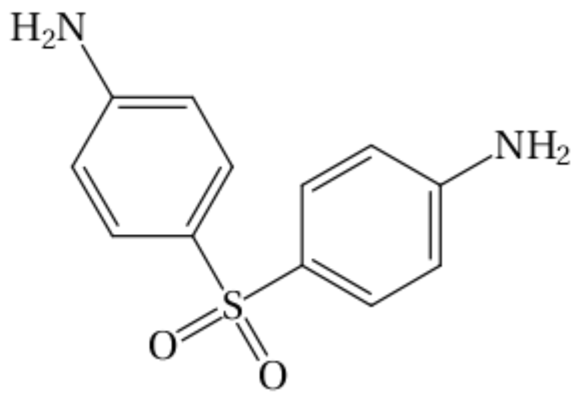

dapsone

Refer to the training data.
What is the aqueous solubility, in logarithmic units of mol/L, of this molecule?

solubility question 7

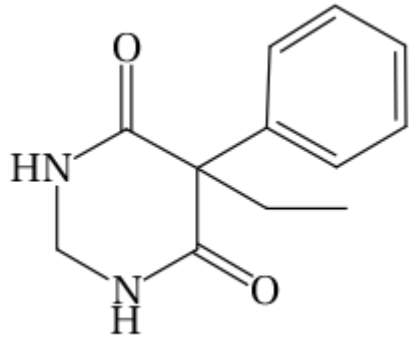

primidone

Refer to the training data.

What is the aqueous solubility, in logarithmic units of mol/L, of this molecule?

solubility question 8

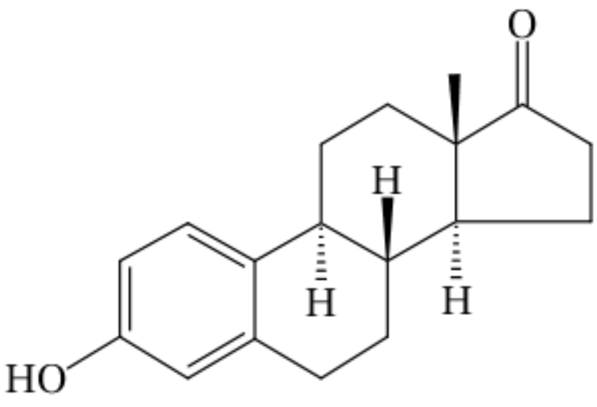

estrone

Refer to the training data.
What is the aqueous solubility, in logarithmic units of mol/L, of this molecule?

solubility question 9

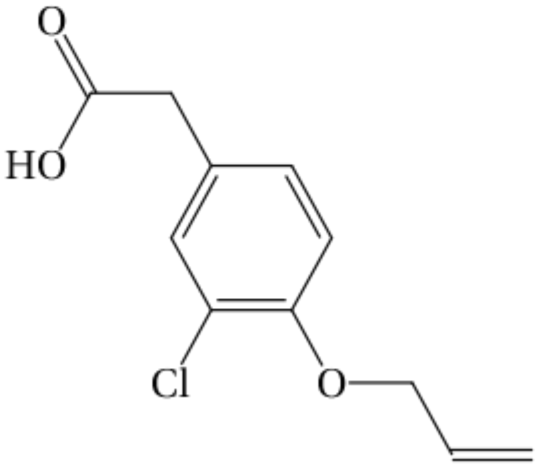

alclofenac

Refer to the training data.
What is the aqueous solubility, in logarithmic units of mol/L, of this molecule?

solubility question 10

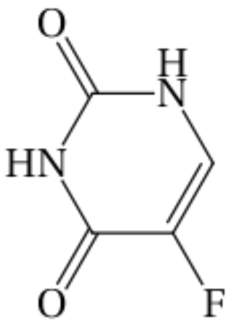

5-fluorouracil

Refer to the training data.
What is the aqueous solubility, in logarithmic units of mol/L, of this molecule?

solubility question 11

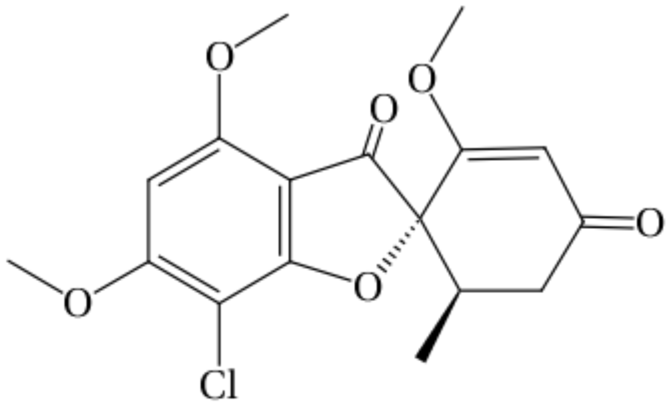

griseofulvin

Refer to the training data.
What is the aqueous solubility, in logarithmic units of mol/L, of this molecule?

solubility question 12

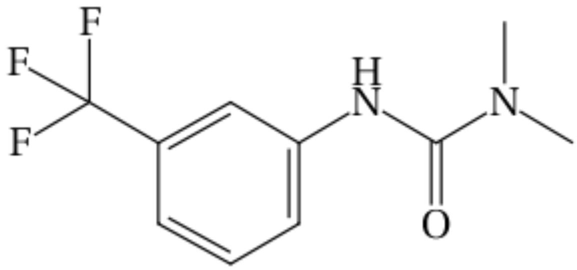

fluometuron

Refer to the training data.
What is the aqueous solubility, in logarithmic units of mol/L, of this molecule?

solubility question 13

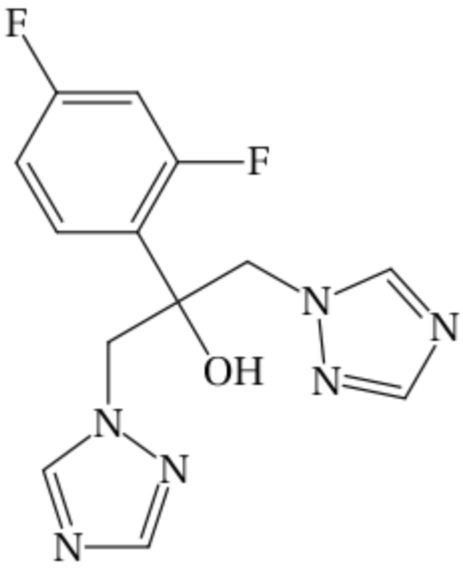

fluconazole

Refer to the training data.
What is the aqueous solubility, in logarithmic units of mol/L, of this molecule?

solubility question 14

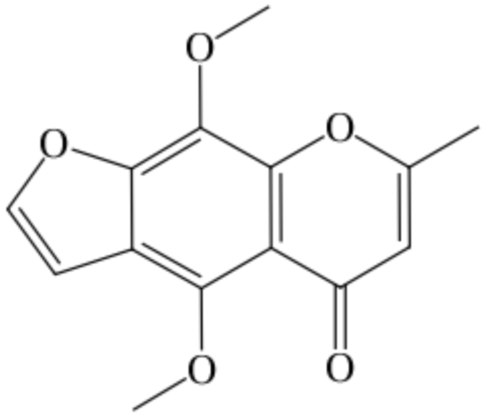

khellin

Refer to the training data.
What is the aqueous solubility, in logarithmic units of mol/L, of this molecule?

solubility question 15

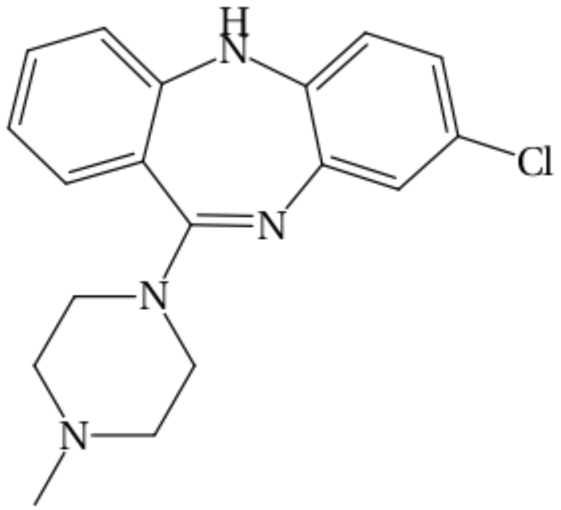

clozapine

Refer to the training data.
What is the aqueous solubility, in logarithmic units of mol/L, of this molecule?

solubility question 16

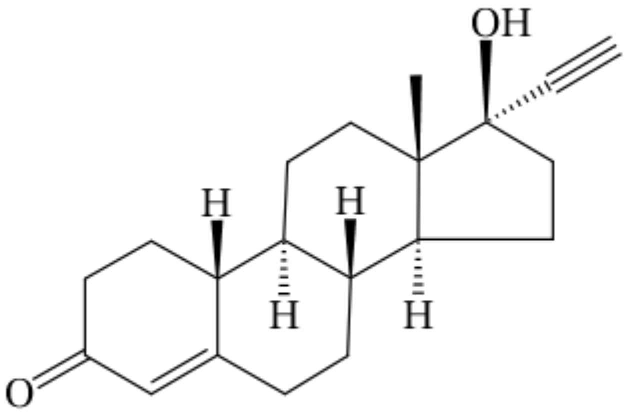

norethisterone

Refer to the training data.
What is the aqueous solubility, in logarithmic units of mol/L, of this molecule?

solubility question 17

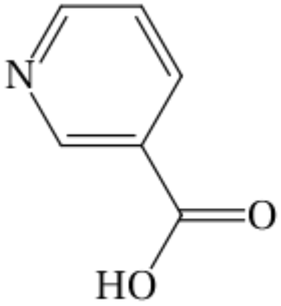

nicotinic acid

Refer to the training data.
What is the aqueous solubility, in logarithmic units of mol/L, of this molecule?

solubility question 18

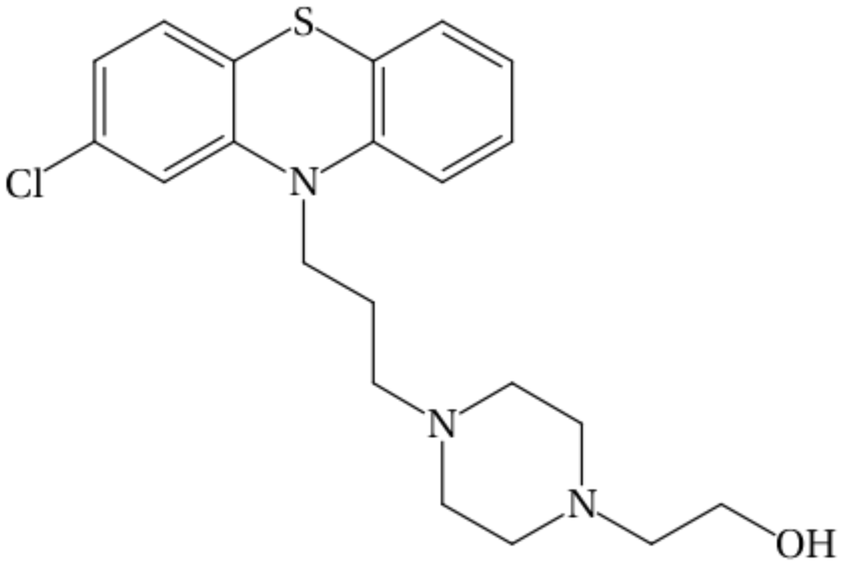

perphenazine

Refer to the training data.
What is the aqueous solubility, in logarithmic units of mol/L, of this molecule?

solubility question 19

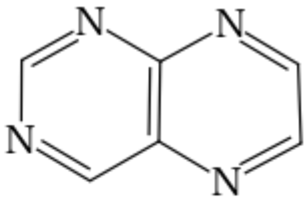

pteridine

Refer to the training data.
What is the aqueous solubility, in logarithmic units of mol/L, of this molecule?

solubility question 20

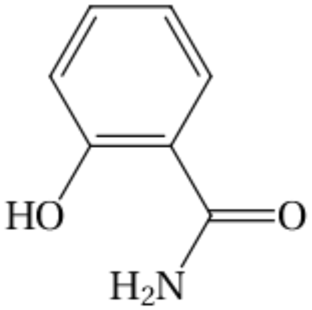

salicylamide

Refer to the training data.
What is the aqueous solubility, in logarithmic units of mol/L, of this molecule?

solubility question 21

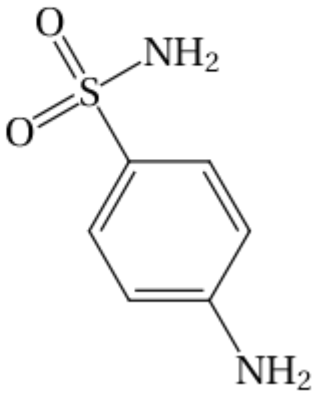

sulfanilamide

Refer to the training data.
What is the aqueous solubility, in logarithmic units of mol/L, of this molecule?

solubility question 22

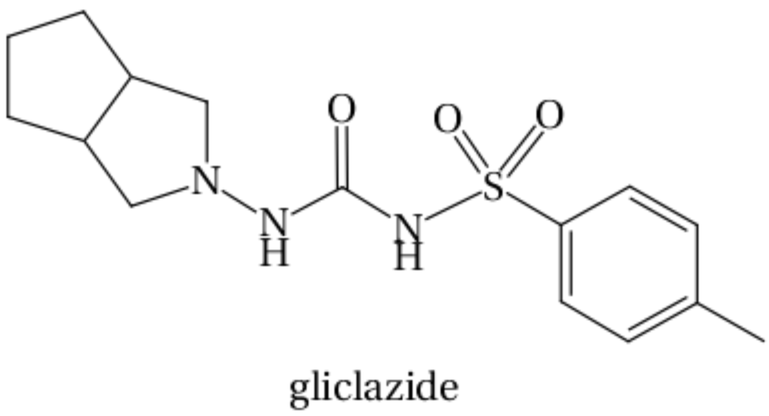

Refer to the training data.
What is the aqueous solubility, in logarithmic units of mol/L, of this molecule?

solubility question 23

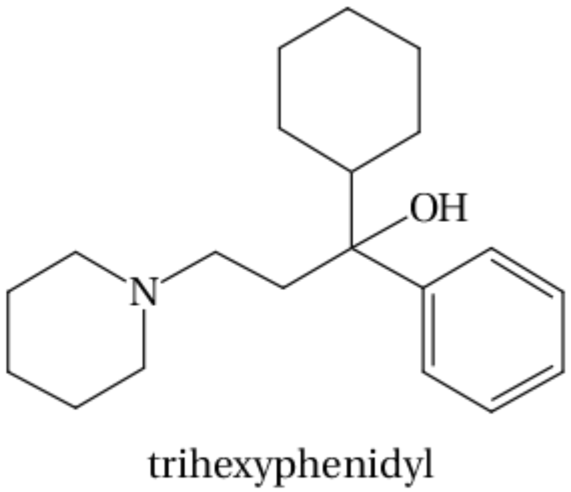

Refer to the training data.
What is the aqueous solubility, in logarithmic units of mol/L, of this molecule?

solubility question 24

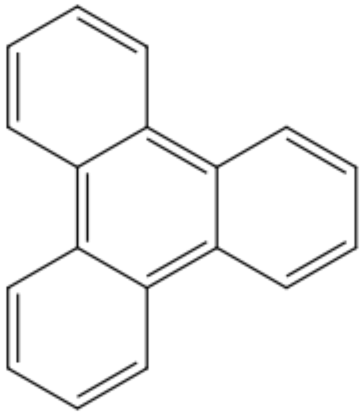

triphenylene

Refer to the training data.
What is the aqueous solubility, in logarithmic units of mol/L, of this molecule?

solubility question 25

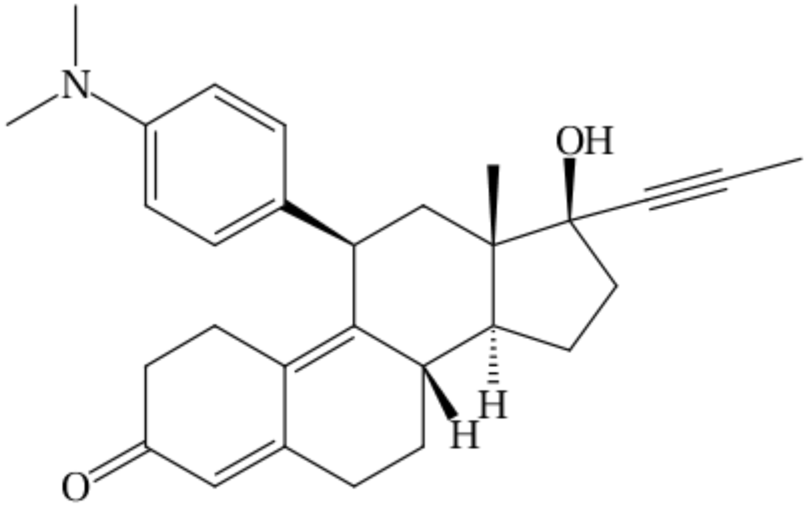

mifepristone

Refer to the training data.
What is the aqueous solubility, in logarithmic units of mol/L, of this molecule?

Debriefing Information

Thank you for taking part in our Solubility Panel exercise. Your help is greatly appreciated.

We are testing the hypothesis that human experts are as good as computation in predicting aqueous solubility of organic molecules. Your responses will be collated and compared to computational methods. We will be using a chemoinformatics-based a machine learning approach to computationally predict solubility. The performance of the participants and computations will be compared and statistically analysed.

We hope to publish the results, but emphasise that no Panel member will be individually identifiable. We will be happy to answer any queries about this project and provide more information (jbom@st-andrews.ac.uk; 01334 467259).

Survey Powered By Qualtrics
